# Edge-based relative entropy as a sensitive indicator of critical transitions in biological systems

**DOI:** 10.1186/s12967-024-05145-3

**Published:** 2024-04-04

**Authors:** Renhao Hong, Yuyan Tong, Huisheng Liu, Pei Chen, Rui Liu

**Affiliations:** 1 School of Mathematics, South China University of Technology, Guangzhou, 510640 China; 2 School of Biology and Biological Engineering, South China University of Technology, Guangzhou, 510640 China

**Keywords:** Critical transition of complex disease, Edge-based relative entropy, Direct interaction networks, Edge-biomarker, Dynamic systems, Informational entropy

## Abstract

**Background:**

Disease progression in biosystems is not always a steady process but is occasionally abrupt. It is important but challenging to signal critical transitions in complex biosystems.

**Methods:**

In this study, based on the theoretical framework of dynamic network biomarkers (DNBs), we propose a model-free method, edge-based relative entropy (ERE), to identify temporal key biomolecular associations/networks that may serve as DNBs and detect early-warning signals of the drastic state transition during disease progression in complex biological systems. Specifically, by combining gene‒gene interaction (edge) information with the relative entropy, the ERE method converts gene expression values into network entropy values, quantifying the dynamic change in a biomolecular network and indicating the qualitative shift in the system state.

**Results:**

The proposed method was validated using simulated data and real biological datasets of complex diseases. The applications show that for certain diseases, the ERE method helps to reveal so-called “dark genes” that are non-differentially expressed but with high ERE values and of essential importance in both gene regulation and prognosis.

**Conclusions:**

The proposed method effectively identified the critical transition states of complex diseases at the network level. Our study not only identified the critical transition states of various cancers but also provided two types of new prognostic biomarkers, positive and negative edge biomarkers, for further practical application. The method in this study therefore has great potential in personalized disease diagnosis.

**Supplementary Information:**

The online version contains supplementary material available at 10.1186/s12967-024-05145-3.

## Introduction

Evidence shows that during the progression of heterogeneous complex disorders, such as various cancer diseases [1, 2], diabetes [3], and epilepsy [4], the deterioration processes are not always steady but occasionally abrupt. Overall, the dynamics of complex disorder development can be considered a nonlinear time-variant dynamical system wherein abrupt deterioration corresponds to a phase change or state transition at a bifurcation point. Thus, the development of diseases typically consists of three stages [5, 6], namely, a before-transition state, a pre-transition/critical state, and an after-transition state (Additional file 1: Fig. S1). Specifically, during a complex disease course, the before-transition state is characterized as a stage with high stability and resilience. The critical state represents the bound of the before-transition state. It is usually reversible and could return to the before-transition state under proper medical interventions, implying the instability of this state [7]; nevertheless, the after-transition state, such as the stage of distant metastasis for cancer, is another steady state with strong irreversibility and resilience after acute deterioration [8]. To achieve the very goal of active prevention, early warning signals should be detected prior to acute disease aggravation; that is, the identification of the critical state throughout the disease course is of crucial importance in predictive and personalized medicine. However, for many complex disorders, it is extremely difficult to identify such a key transition due to few phenotypic distinctions between the before-transition state and the pre-transition state and a lack of effective universal disease models [9].

Great efforts have been devoted to discovering biomarkers for better diagnosing the after-transition state. Based on the differential expression of genes/nodes, many important molecular biomarkers with consistently high/low expression in the deteriorated state, such as *BRCA1* and *TP53*, were found to be effective in indicating the development of breast cancer and lung cancer [10, 11]. However, the onset of complex diseases usually arises from the dysregulation of signaling functions and/or the cell’s response to its microenvironment, which are driven by dynamic changes in complex interactions among many molecules or molecular modules rather than individual molecules [12]. In fact, intermolecular interactions, considered the edges of biological networks, constitute the foundation of and facilitate biological functions and signal transmission [13]. Hence, it is important and necessary to explore the dynamic change in biomolecular interactions of biological systems, thus identifying the tipping points or critical transitions in complex diseases by providing a comprehensive understanding of the biomolecular network. In addition, some studies suggest that subtle changes in some non-differentially expressed genes (non-DEGs) can also have significant biochemical consequences, thereby playing an important role in various biological functions [14, 15]. Thus, methods based on networks that explore differential intermolecular interactions/edges rather than differential expression/nodes may better characterize the development of complex diseases before catastrophic deterioration [16]. Edge biomarkers, as a type of promising network biomarker, may reveal the underlying mechanism of dynamic changes in molecular associations or regulatory relationships and provide a comprehensive perspective for understanding complex disease pathogenesis from a network standpoint.

**Fig. 1:**
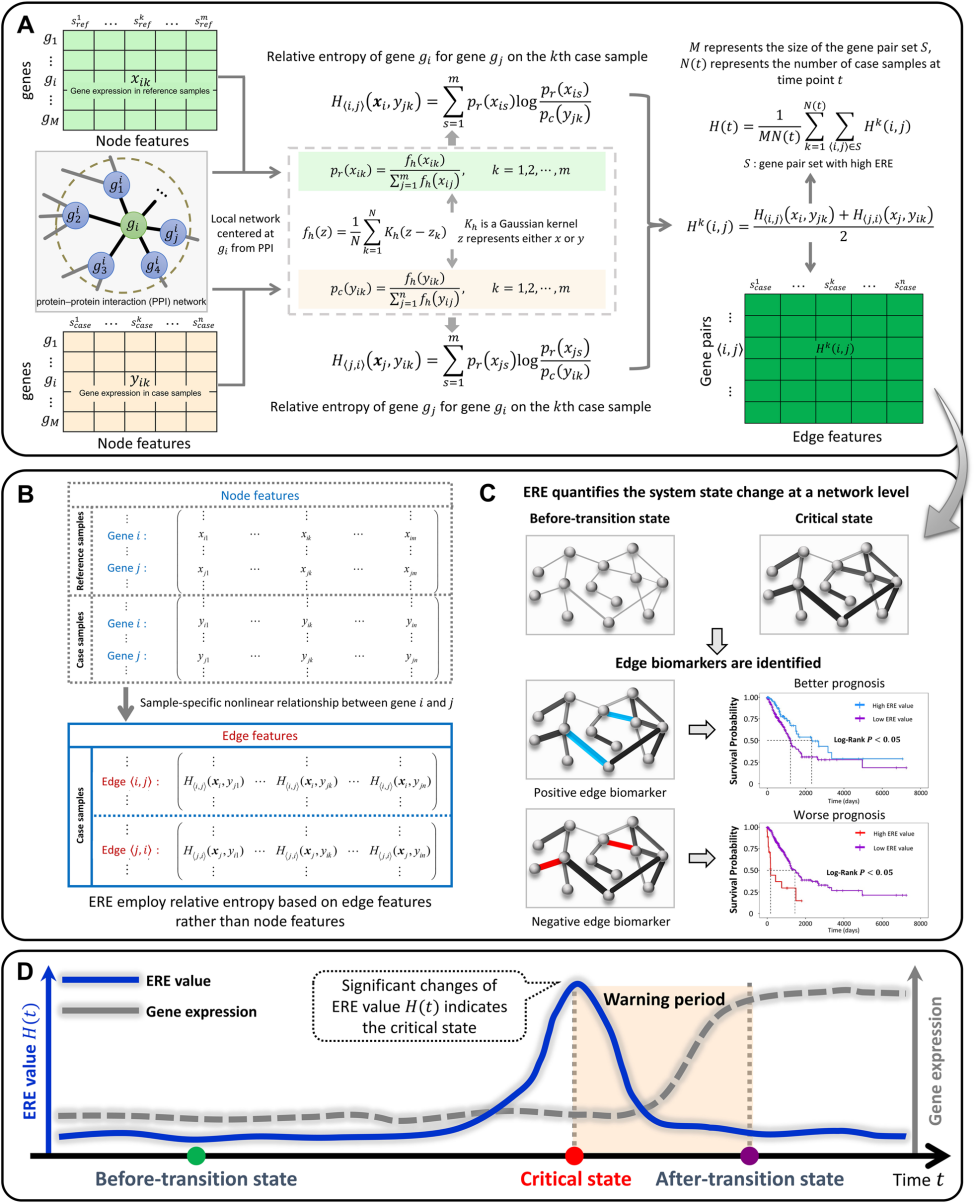
Schematic diagram of the edge-based relative entropy (ERE) method. **A** Given reference samples from a relatively healthy cohort and case samples to be tested, the probability corresponding to the expression of each gene in an individual sample under the two conditions is calculated separately using kernel density estimation (KDE). The entropy matrix is then obtained. **B** Matrices regarding node and edge features. **C** During the progression of complex diseases, ERE can effectively distinguish the before-transition and pre-transition states at the network level and identify some edge biomarkers for prognosis analysis. **D** The significant change in ERE may indicate a critical state of a complex disease

In this study, we propose a model-free method based on edge-based relative entropy (ERE) to identify early warning signals of disease deterioration (Fig. 1). The ERE method is theoretically based on the framework of dynamic network biomarkers [17]. By considering the combination of intergenic associations and assigning samples accordingly (Fig. 1A), the ERE method constructs a vector form of edge-based relative entropy values that can be viewed as the “edge feature” to represent the interaction information between each pair of variables/nodes (Fig. 1B), which can be employed to measure the similarity between probability distributions of the corresponding two genes in high-dimensional nonlinear biosystems. In this way, ERE transforms the gene expression matrix (with only node information) into an entropy matrix (with information on gene associations and networks) and offers a quantitative way to identify whether the to-be-tested/case samples are derived from a critical state. Therefore, those pairs of molecules (or edges) can be identified from molecular interactions with high ERE values (Fig. 1C), thus serving as edge biomarkers that help to signal the critical transition in biological processes (Fig. 1D). Furthermore, these edge biomarkers can be categorized into two types according to corresponding disease outcomes, such as the prognosis of patients, that is, positive/negative edge biomarkers indicating good/poor prognosis. Moreover, it is possible to uncover some “dark genes” that are non-differential at the expression level but the components of important gene interactions involved in key biological functions. Compared to the other algorithms representing the edge information using linear methods [18], *e.g.*, Pearson correlation coefficient (PCC), the ERE method elucidates the sample-specific nonlinear relationship between a pair of molecules/genes, which optimizes the identification ability with strong robustness. Clearly, the proposed ERE method is of high applicability and can be utilized with any molecular network structure. To demonstrate the validity of the proposed method, the ERE method was applied to a simulated dataset and six real datasets, including colon adenocarcinoma (COAD), lung adenocarcinoma (LUAD), thyroid adenocarcinoma (THCA), kidney renal clear cell carcinoma (KIRC) and kidney renal papillary cell carcinoma (KIRP) datasets from The Cancer Genome Atlas (TCGA) database and an acute lung injury dataset (GSE2565) from the Gene Expression Omnibus (GEO) database. The critical states ahead of severe clinical deterioration were discriminated in the different stages of tumors. The identified critical states were all coincident with experimental observations or survival analysis. Some of the edge biomarkers were also verified by a series of functional enrichment analyses and prognosis analyses. In summary, the ERE method may provide a reference computational method and quantitative indicator for biomedical studies, and positive/negative edge biomarkers identified based on this method may be clinical early warning signs for diseases.

## Materials and methods

### The ERE algorithm

Given $m$ reference samples from a comparatively healthy cohort that represent individuals in a healthy (relatively healthy) state and $n$ case samples to be tested, we identify the critical state by carrying out the following procedures:

1. Mapping of the gene expression to the network structure. For gene ${g}_{i}$, denote the expression values in the reference set as $({x}_{i1},{x}_{i2},...,{x}_{im})$ and the expression values of case samples as $({y}_{i1},{y}_{i2},...,{y}_{in})$.
2. Fitting of probability vectors for each gene/node, which was based on the reference and case samples (Fig. 1A). Specifically, for a gene ${g}_{i}$, the probability ${p}_{r}\left({x}_{ij}\right)$ is calculated based on the reference samples $({x}_{i1},{x}_{i2},...,{x}_{im})$ as follows:
  $${p}_{r}\left({x}_{ik}\right)=\frac{{f}_{h}\left({x}_{ik}\right)}{\sum_{j=1}^{m}{f}_{h}\left({x}_{ij}\right)}, k=\mathrm{1,2},\dots ,m.$$
  Here, the probability density estimator ${f}_{h}\left(z\right)$ is defined as
  1 $${f}_{h}\left(z\right)=\frac{1}{N}\sum_{k=1}^{N}{K}_{h}\left(z-{z}_{k}\right)=\frac{1}{Nh}\sum_{k=1}^{N}K\left(\frac{z-{z}_{k}}{h}\right),$$
  where $K$ denotes a nonnegative Gaussian kernel function, and bandwidth $h ={\left(\frac{4{\sigma }^{5}}{3N}\right)}^\frac{1}{5}> 0$ denotes a smoothing parameter with $\sigma$ as the standard deviation of samples and $N$ as the number of samples (see Additional file 1: Note S1 for details). Note that KDE excels at estimating unknown distributions from empirical data, accommodating irregular structures without the need for understanding underlying processes. But the values of bandwidths in KDE are calculated from the observed data reflecting the true state of the system. Therefore, we only focus on the sampled points when calculating the ERE values. Clearly, vector ${P}_{r}=({p}_{r}\left({x}_{i1}\right),{p}_{r}\left({x}_{i2}\right),...,{p}_{r}\left({x}_{im}\right))$ satisfies $\sum_{j=1}^{m}{p}_{r}\left({x}_{ij}\right)=1$, and ${p}_{r}\left({x}_{ij}\right)>0$. Similarly, the probability vector ${P}_{c}=({p}_{c}\left({y}_{i1}\right),{p}_{c}\left({y}_{i2}\right),...,{p}_{c}\left({y}_{in}\right))$ can be calculated based on the case samples $({y}_{i1},{y}_{i2},...,{y}_{in})$, with
  $${p}_{c}\left({y}_{ik}\right)=\frac{{f}_{h}\left({y}_{ik}\right)}{\sum_{j=1}^{n}{f}_{h}\left({y}_{ij}\right)}, k=\mathrm{1,2},\dots ,n.$$
3. Calculation of the ERE value of each case sample by using the edges in the protein–protein interaction (PPI) network. Specifically, for a pair of associated genes ${g}_{i}$ and ${g}_{j}$ in the $k$ th case sample,
  2 $${H}_{\langle j,i\rangle }\left({{\varvec{x}}}_{j},{y}_{ik}\right)={p}_{r}\left({x}_{j1}\right){\text{log}}\frac{{p}_{r}\left({x}_{j1}\right)}{{p}_{c}\left({y}_{ik}\right)}+{p}_{r}\left({x}_{j2}\right){\text{log}}\frac{{p}_{r}\left({x}_{j2}\right)}{{p}_{c}\left({y}_{ik}\right)}+\dots +{p}_{r}\left({x}_{jm}\right){\text{log}}\frac{{p}_{r}\left({x}_{jm}\right)}{{p}_{c}\left({y}_{ik}\right)},$$
  where ${y}_{ik}$ represents the expression values of ${g}_{i}$ in the $k$ th case samples. In general, ${H}_{\langle i,j\rangle }\left({{\varvec{x}}}_{i},{y}_{jk}\right)\ne {H}_{\langle j,i\rangle }\left({{\varvec{x}}}_{j},{y}_{ik}\right)$. In this study, we use the following symmetric measure:
  3 $${H}^{k}\left(i,j\right)=\frac{{H}_{\langle i,j\rangle }\left({{\varvec{x}}}_{i},{y}_{jk}\right)+{H}_{\langle j,i\rangle }\left({{\varvec{x}}}_{j},{y}_{ik}\right)}{2},$$
  which indicates the local ERE value calculated from the gene pair ${g}_{i}$ and ${g}_{j}$ for the $k$ th case sample. For the $k$ th case sample, we calculate the sample-specific ERE value ${H}^{k}$ according to a crowd of gene pairs with the highest ERE values, *i.e.*, ${H}^{k}=\frac{1}{M}\sum_{\langle i,j\rangle \in S}{H}^{k}(i,j)$, where $\langle i,j\rangle$ represents the gene pair ${g}_{i}$ and ${g}_{j}$, $S$ is the high-ERE value (top 5% by default) gene pair set in the $k$ th case sample and constant $M$ is set as the size of $S$ for this study.

At each time point $t$, the ERE value $H(t)$ is calculated based on the above procedures, with $H(t)= \frac{1}{N(t)}\sum_{k=1}^{N(t)}{H}^{k}(t)$, where $N(t)$ represents the case sample size at time point $t$. The effective signal is identified through the one-sample *t*-test, which is presented in Additional file 1: Note S3. Specially, when $t=2$, the time point $T=t$ is considered a critical point if $H(t)$ is significantly different from the mean of vector $(H(1),H(3))$.

### Data processing and functional analysis

ERE was applied to six sets of gene expression data, *i.e.*, the cancer datasets of COAD, LUAD, THCA, KIRC and KIRP from the TCGA database and the time-course dataset of acute lung injury (GSE2565) from the NCBI GEO database. Concerning the microarray data (GSE2565), we only reserved the probes with corresponding gene symbols and employed the mean value of multiple probes for the same gene as the expression level of the mapped gene. Each of the cancer datasets includes tumor-adjacent and tumor samples. The tumor-adjacent samples were utilized as reference samples. The tumor or case samples were screened and partitioned according to the corresponding clinical information (Table 1).

**Table 1:** The number of samples in each tumor stage in each TCGA dataset

Types of cancer	TA samples	Stage I		Stage II		Stage III			Stage IV
		Stage IA	Stage IB	Stage IIA	Stage IIB	Stage IIIA	Stage IIIB	Stage IIIC	
COAD	42	72		154	11	28	55	37	62
LUAD	59	162		29	39	51	7	0	19
THCA	58	268		52		109			53
KIRC	72	199		39		63			51
KIRP	32	160		19		45			14

TA samples: tumor-adjacent samples

The pathway enrichment analysis was carried out through the clusterProfiler package [19] and the Kyoto Encyclopedia of Genes and Genomes (KEGG) (https://www.genome.jp/kegg/). Survival analysis was carried out on the basis of Kaplan–Meier log-rank analysis. The PPI networks of *Homo sapiens* and *Mus musculus* were constructed based on information from the Search Tool for the Retrieval of Interacting Genes/Proteins (STRING, http://string-db.org).

## Results

The definition of the ERE value and its algorithm are presented in the Methods section. To demonstrate the effectiveness of ERE, it was tested on a simulated sixteen-node dataset (see Additional file 1: Notes S2 and S9 for details) and applied in six real datasets, including acute lung injury (GSE2565) from the GEO database and COAD, LUAD, THCA, KIRC and KIRP from the TCGA database. The successful identification of the critical state in the complex disease progression verified the applicability of our method in quantitatively identifying the tipping point ahead of irreversible deterioration of health. In this process, some edges with high entropy in the critical state were selected as signaling edges for in-depth analysis.

### Identifying the critical transition in acute lung injury

**Fig. 2:**
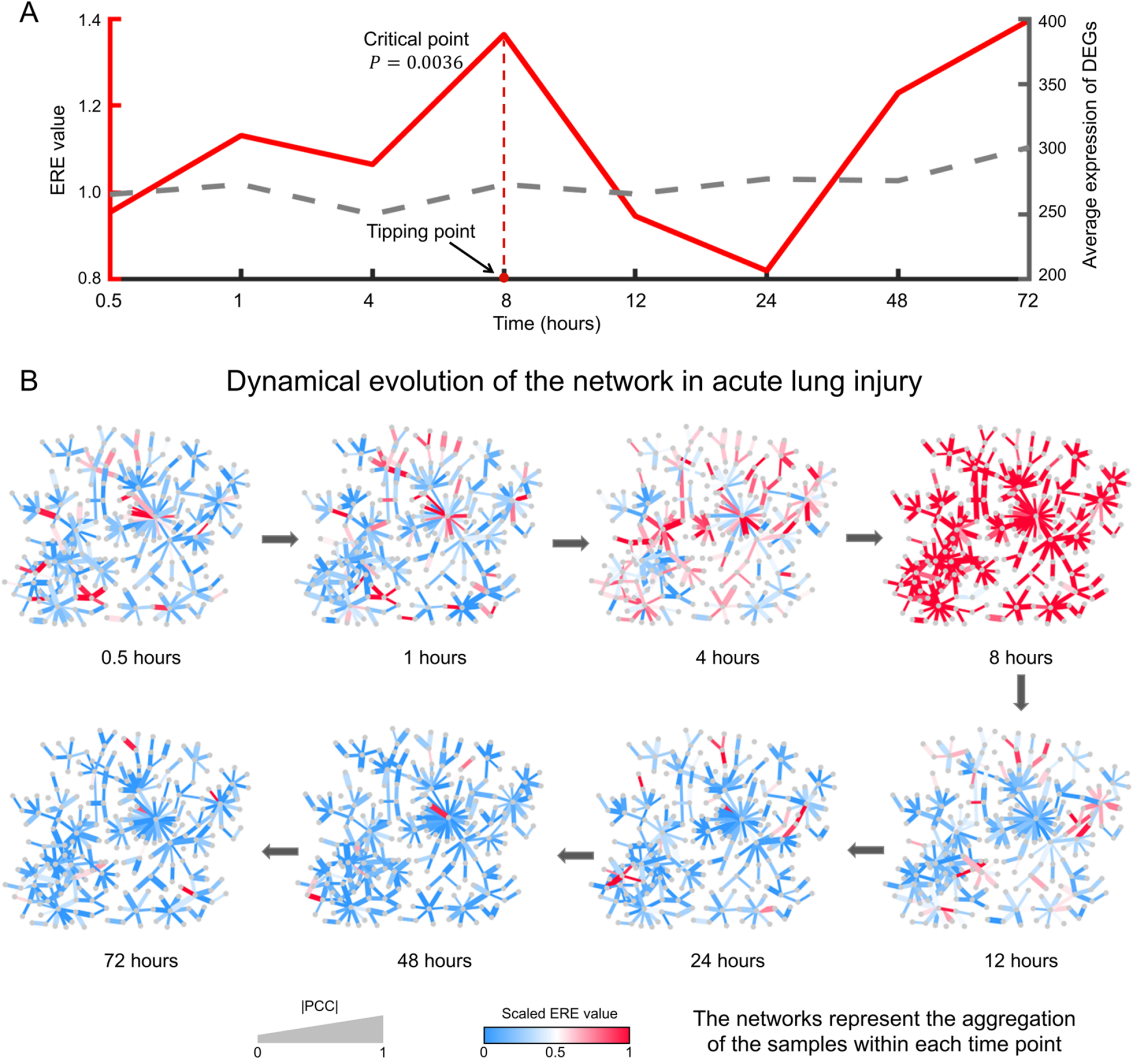
Performance of ERE in acute lung injury. **A** Performance comparison of ERE and DEGs in identifying the critical state. **B** The dynamic evolution of the molecular network consisting of the signaling edges (with the top 5% ERE values) revealed a significant change in the network at 8 h. The networks represent the aggregation of ERE values from all the samples within each time point

ERE was applied to the microarray gene expression data of mice obtained from an experiment of phosgene-induced acute lung injury [20]. The control and case samples were generated by exposing two sets of mice to air or phosgene, respectively. Subsequently, lung tissues from air- or phosgene-exposed mice were collected at 0.5, 1, 4, 8, 12, 24, 48, and 72 h. It was found that a 50–60% death rate in the case group was observed after 12 h, while there was a 60–70% mortality rate after 24 h. Notably, the most deadly acute lung injury caused by phosgene occurred approximately 12 h after exposure [20]. For the case group, the ERE value sharply increased from 4 to 8 h after exposure (Fig. 2A), implying a correspondence between the critical state and the 4-h time window from 4 to 8 h, with the system entering the after-transition state after the 8-h point. However, the average normalized expression of differentially expressed genes (DEGs) fails to signal the forthcoming system state transition (Fig. 2A). The computational results agree with the experimental observations, suggesting the effectiveness of the ERE method in the biological experiment. More details describing the variability of the ERE value at each time point and the expression calculation of DEGs are provided in Additional file 1: Fig. S19 and Note S10.

### Identifying the critical states for various cancers

**Fig. 3:**
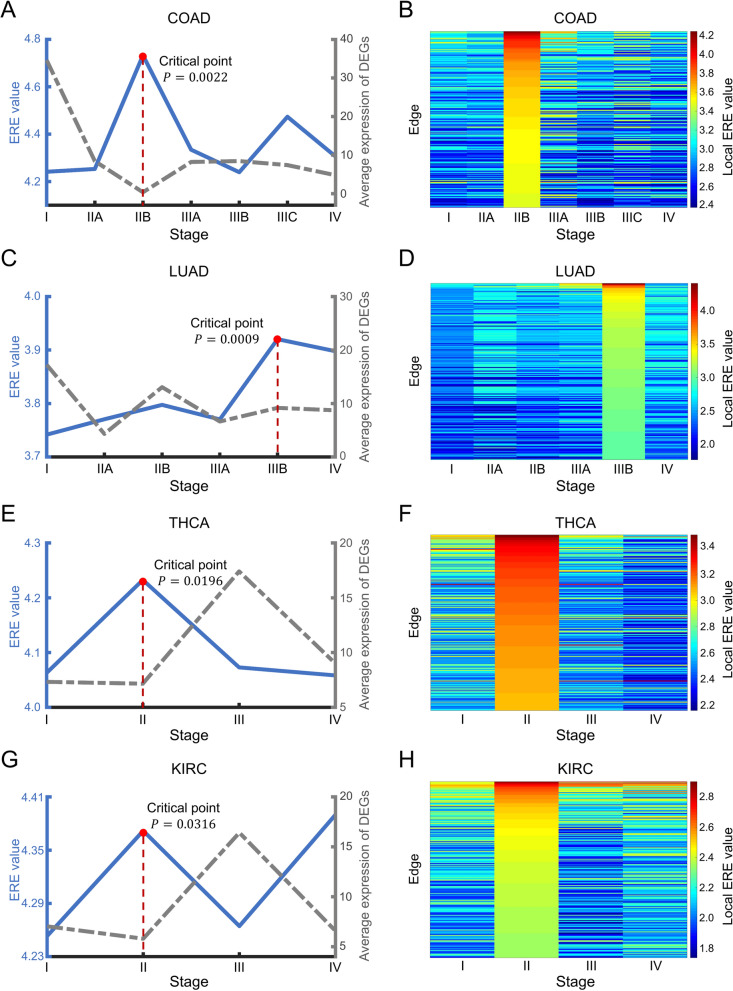
Identifying critical states for tumor deterioration. The performance comparison of ERE and DEGs in identifying the critical states for different tumor datasets: **A** COAD, **C** LUAD, **E** THCA, and **G** KIRC. The local ERE values of the high-entropy edges in the identified critical stages are depicted as two-dimensional heatmaps across all stages for each dataset: **B** COAD, **D** LUAD, **F** THCA, and **H** KIRC

Then, ERE was applied to five TCGA datasets: COAD, LUAD, THCA, KIRC and KIRP (Additional file 1: Fig. S2). Implementing the procedure in the Materials and Methods, we obtained the ERE value for each individual tumor sample. Then, the average ERE value at every stage was calculated and visualized for the identification of the critical state (Fig. 3).

By applying the proposed method, the significant increase in the ERE value identified the critical states for four common cancers, *i.e.,* stage IIB ($P=0.0022$) for COAD (Fig. 3A), stage IIIB ($P=0.0009$) for LUAD (Fig. 3C), stage II ($P=0.0196$) for THCA (Fig. 3E), and stage II ($P=0.0316$) for KIRC (Fig. 3G). Clearly, the mean expression of DEGs and traditional gene biomarkers (Additional file 1: Table S1 and Fig. S11) cannot indicate such critical transitions. The heat maps of local ERE values for the four cancers (Fig. 3B, D, F, H) illustrate that the local ERE values of signaling edges increase drastically in a collective manner at the identified critical states during disease progression. The computational results are consistent with the clinical observations. Specifically, at the identified critical state (stage IIB) of COAD, the cancer has not yet metastasized, whereas at stage IIIA, it has already spread to the nearby lymph nodes [21]. At the identified critical state of LUAD (stage IIIB), the metastasis of cancer has not occurred, whereas cancer cells enter distant tissues or organs through the bloodstream at stage IV [22]. For THCA, metastasis has not occurred yet at the identified critical state (stage II); nevertheless, regional lymph node metastasis occurs at stage III [23]. For KIRC, the tumor is noninvasive at the identified critical state (stage II), whereas at stage III, tumor cells spread to surrounding tissues [24]. Additional file 1: Fig. S11 B, D, F, and H show that the survival expectancy is much higher before the identified critical state than afterward. Moreover, the prognosis analysis also supports the computational results based on ERE. For example, the difference in survival expectancy before and after the identified critical state of THCA, *i.e.*, stage II, is the most significant ($P<0.0001$) compared with the prognosis analysis based on other stage divisions (see Additional file 1: Fig. S13 for details of the prognosis analysis).

**Fig. 4:**
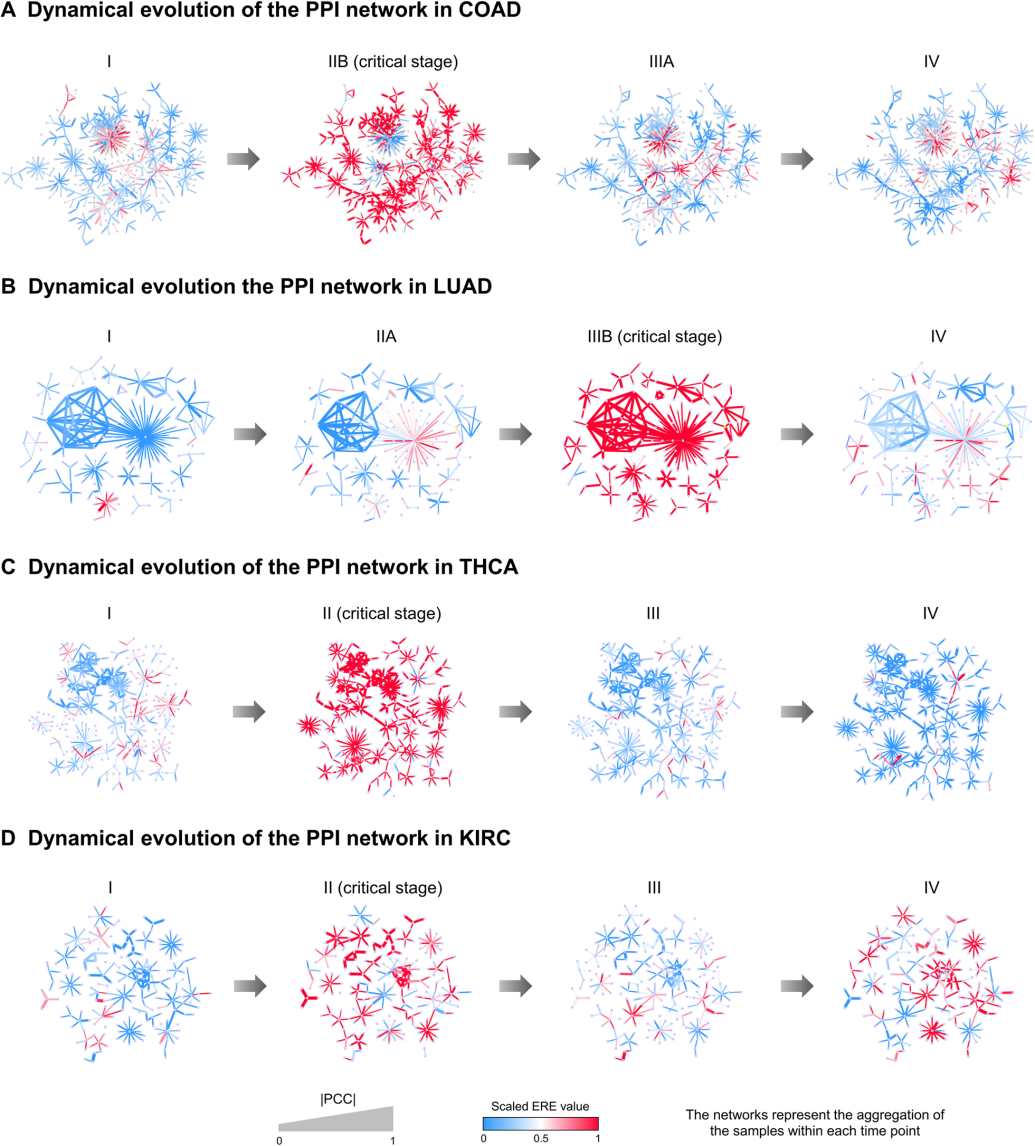
Dynamic evolution of networks consisting of ERE signaling edges in COAD, LUAD, THCA, and KIRC. **A** In COAD, the subnetwork composed of ERE signaling gene pairs evolved, with a clear distinction between stage IIB and other stages. **B** Similarly, there were clear changes in the subnetworks at stage IIIB for LUAD. **C** The subnetwork in THCA showed abrupt changes at stage II. **D** The subnetwork in KIRC also showed dramatic changes at stage II. The networks represent the aggregation of ERE values from all the samples within each time point

In addition, the PPI network composed of high-ERE value gene pairs (top 5% in the critical stage) helps us understand the dynamic changes in the ERE signaling edges at a network level. Drastic changes in the PPI networks occurred at stage IIB of COAD (Fig. 4A), stage IIIB of LUAD (Fig. 4B), stage II of THCA (Fig. 4C), and stage II of KIRC (Fig. 4D), suggesting the following catastrophic deterioration for each disease. Notably, there are some essential cancer-related hub genes captured in the above network, such as *GATA4*, which is well known for its antitumor function in COAD [25]. More details for the networks in each cancer are provided in Additional file 1: Table S3 and Fig. S12.

### Positive and negative edge biomarkers

**Fig. 5:**
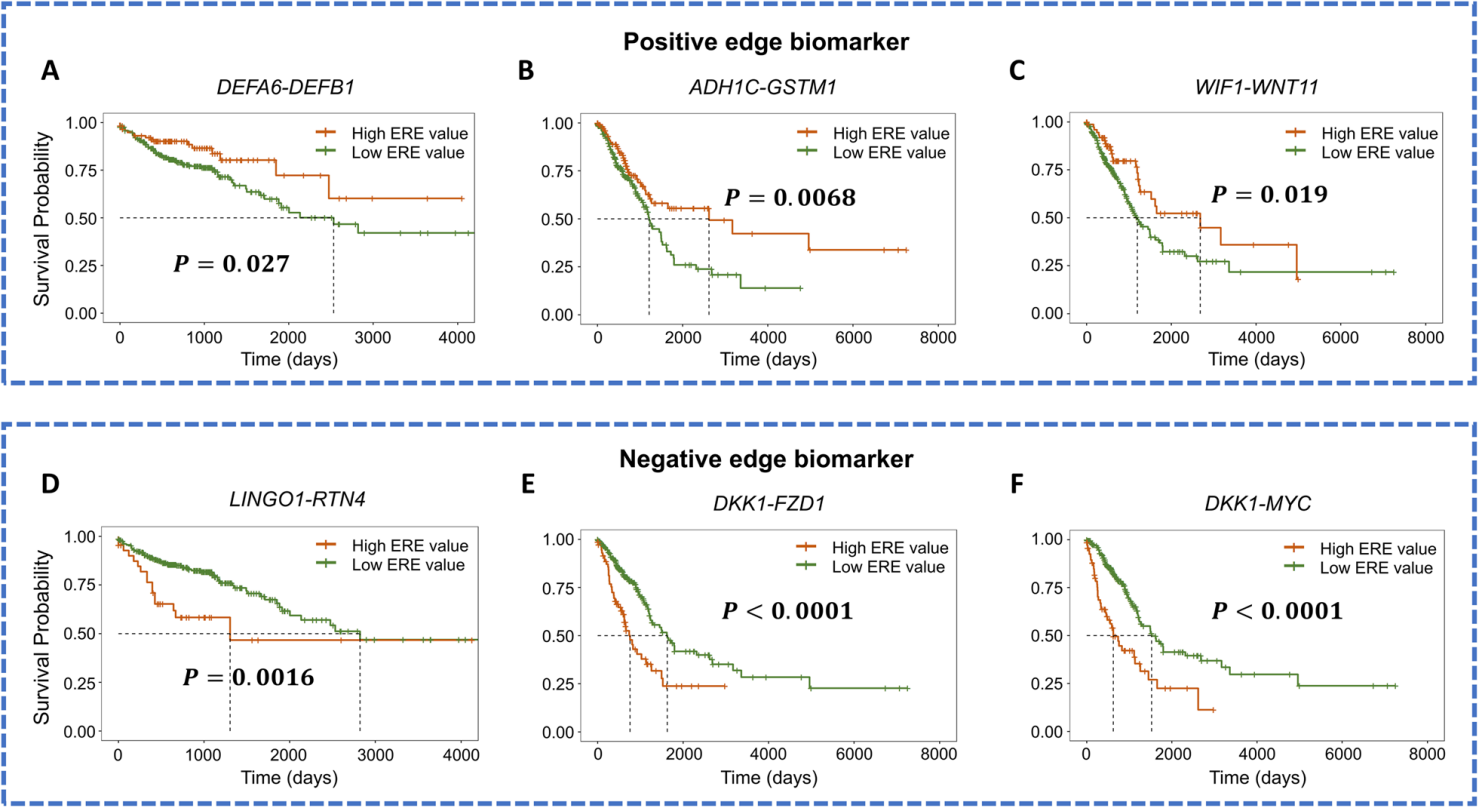
Survival analysis based on positive and negative edge biomarkers. **A**–**C** The survival expectancy of patients with high ERE values for positive edge biomarkers is significantly longer than those with low ERE values for the biomarkers. **D**–**F** The survival expectancy of patients with high ERE values for negative edge biomarkers is significantly shorter than those with low ERE values for the biomarkers

Based on the clinical information, the samples were classified into a long-survival group (with a long survival expectancy, *i.e.*, more than 5 years) and a short-survival group (with a short survival expectancy, *i.e.*, less than 5 years). If an edge presented a high ERE value in over 80% of samples of the long-survival/short-survival group, it was defined as a positive/negative edge biomarker, as shown in Fig. 5A–F for three positive/negative edge biomarkers. These edge biomarkers quantitatively identify the critical states during disease progression as signaling gene pairs and are also effective for analyzing the prognosis of cancer. Taking LUAD as an example, the survival expectancy of patients with high ERE values for the positive edge biomarker *ADH1C-GSTM1* was significantly longer ($P=0.0068$) than that of patients with low ERE values for the biomarker (Fig. 5B). Furthermore, edge biomarkers may exert important regulatory effects on disease progression from the perspective of cancer-related signaling pathways. For example, *EGFR*-*MYC* and *EGFR-RAC1* were identified as negative edge biomarkers for LUAD. By KEGG pathway enrichment analysis, they were found to be enriched in the MAPK signaling pathway, which is an essential signaling cascade in the growth and development of tumor cells [26]. More details about the edge biomarkers for the four cancers are shown in Additional file 1: Table S4, Fig. S8 and Note S4.

### Revealing non-differentially expressed “dark genes” and potential signaling mechanisms via the ERE method

The ERE-based analysis suggests that some non-DEGs may be regarded as “dark genes” and exert important functions in disease progression and prognosis analysis (Table 2). For example, *DKK1* was non-differentially expressed but could distinguish the prognosis of LUAD patients as the component of an edge (*DKK1-FZD1*) based on the ERE method. The results of KEGG enrichment analysis illustrated that these “dark genes” were closely related to cancer development (Additional file 1: Table S2).

**Table 2:** Dark genes as components of edge biomarkers in LUAD and KIRC

Gene	Associated edge biomarker	Type	Location	Family	Relation with tumors
*DKK1*	*DKK1-FZD1*	Negative	Extracellular	Other	*DKK1* guides epithelial-mesenchymal transition and promotes non–small cell lung cancer (NSCLC) invasion and metastasis [30]
*MYC*	*DKK1-MYC*	Negative	Nucleus	Transcription factor	*MYC* functions as a metastasis promoter for NSCLC [31]
*WNT1*	*DKK1-WNT1*	Negative	Plasma membrane	Other	*WNT1* is closely related to tumor proliferation and angiogenesis in NSCLC [32]
*GAPDH*	*GAPDH-MYC*	Negative	Cytosol	Enzyme	*GAPDH* is related to the proliferation and migration of lung cancer [33]
*GNG4*	*GNB1-GNG4*	Negative	Plasma membrane	Other	*GNG4* may promote the proliferation and metastasis of cancer cells by affecting their EMT progression [34]
*EGFR*	*EGFR-MYC*	Negative	Endosome	Protein kinase	*EGFR* mutations usually result in tumor cellular proliferation in lung cancer [35]
*FOSL1*	*FOSL1-MYC*	Negative	Nucleus	Transcription factor	*FOSL1* likely exerts essential functions in tumor growth and metastasis [36]
*NOG*	*BMP7*-*NOG*	Negative	Extracellular	Other	*NOG* may inhibit tumor- suppressing properties of the BMPs and cause tumorigenesis [37]

**Fig. 6:**
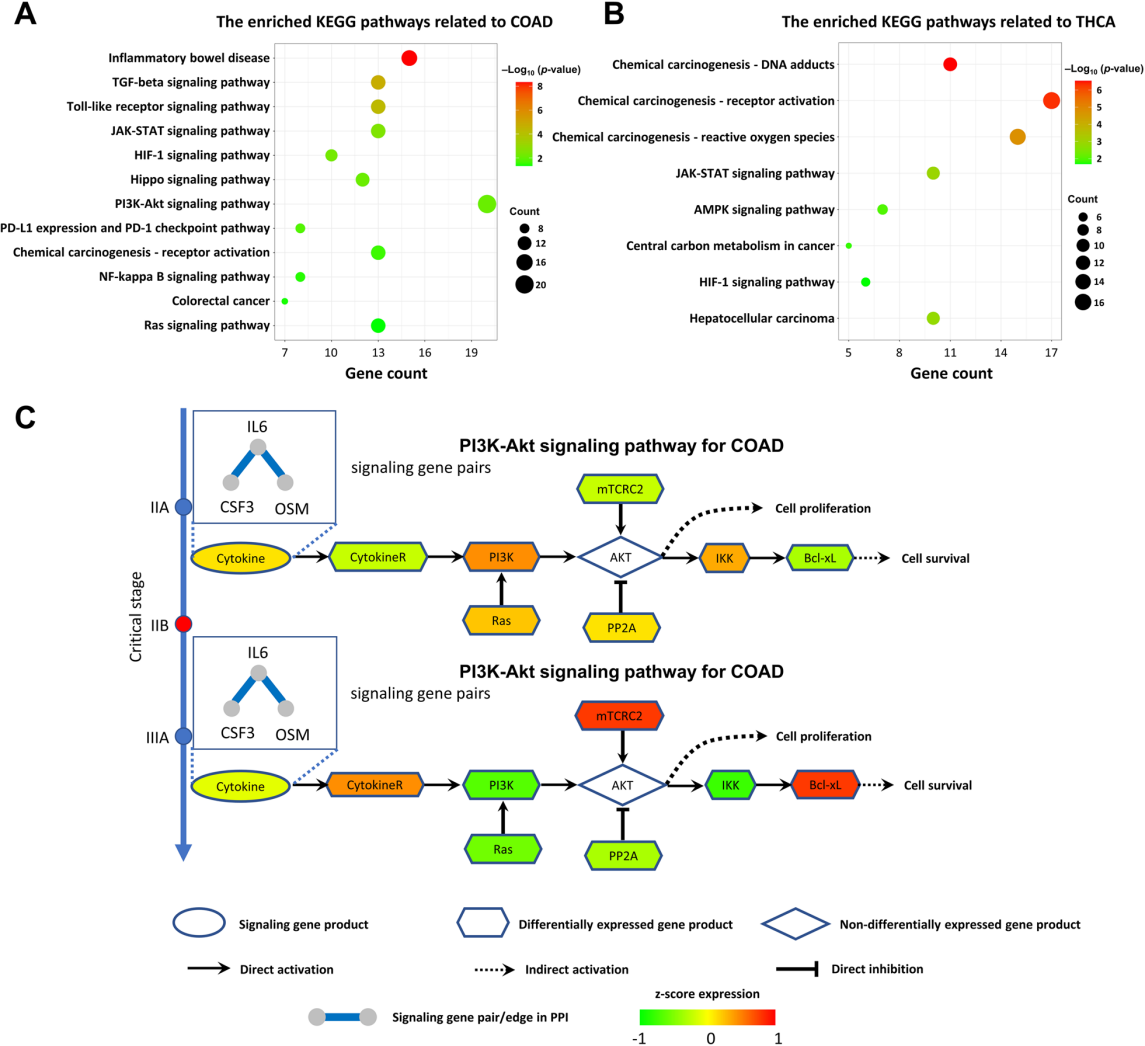
The regulatory mechanism of cancer development revealed by ERE signaling gene pairs. KEGG pathway enrichment analysis for the ERE signaling gene pairs of **A** COAD and **B** THCA. **C** Switching dynamics of downstream differential genes before and after the critical state conducted by upstream ERE signaling gene pairs in COAD. Cytokines are coded by *IL6*, *CSF3*, and *OSM*, which correspond to signaling gene pairs after mapping into the PPI network

Tumor progression is a process of dysfunctional changes [27]. To further explore the functional relevance of signaling gene pairs and tumor progression, we performed pathway enrichment analysis for signaling gene pairs. As illustrated in Fig. 6A and B, the gene pairs were mainly enriched in some classic cancer-relevant pathways, such as the TGF-β and JAK-STAT signaling pathways, for COAD and THCA, respectively (see Additional file 1: Table S2 for details). These pathways are involved in cell proliferation and migration, angiogenesis, immune changes and metastasis in tumor progression [28, 29].

To further investigate how signaling genes affect alterations in gene expression before and after the pre-transition state in the PI3K-Akt signaling pathway, the underlying molecular mechanism was unraveled based on the functional analysis of the COAD signaling gene pairs, as shown in Fig. 6C. In stage IIA and earlier stages, tumor cells were disordered and might have prompted critical transitions by cytokines (signaling genes), such as *IL6*, *CSF3* and *OSM*, in the microenvironment. After the critical stage (stage IIB), signaling receptor genes (*IL4R*) were highly expressed, which might have triggered the phosphorylation of PI3K and AKT proteins and further upregulated the expression of the apoptosis inhibitor *BCL2L1* and enhanced tumor cell growth and proliferation. Overall, ERE signaling gene pairs affected the PI3K/AKT signaling pathway in tumor progression and were involved in many cancer-related pathways (for details, see Additional file 1: Fig. S7 and Notes S6 and S7).

## Discussion

Early diagnosis is helpful to prevent the development of severe disease. Therefore, it is of vital importance to detect early warning signals before catastrophic deterioration. Nevertheless, disease progression usually results from dynamic changes in complex interactions among many molecules rather than individual molecules. The shortcomings of conventional node-based methods are becoming apparent when they differentiate the critical state from the before-transition state due to their static nature, where molecules exhibit limited expression changes from the before-transition state to the critical state. The previous DNB method, which is based on genes/nodes and neglects edges/gene‒gene associations or network structure, employs the fluctuation (*i.e.*, the standard deviation) and covariance of samples to identify the tipping point of the disease process (see Additional file 1: Note S8 for details). In addition, the DNB method necessitates a balanced number of control group samples and case group samples at each time point, which proves to be exceedingly challenging to achieve in practical biomedicine. In this study, from a network analysis standpoint, we propose a computational method to quantify the dynamic changes in the cooperative effects of biomolecular interactions, thus effectively signaling the tipping points during disease progression. The proposed ERE method is different from the previous DNB method in the following two aspects. First, our approach detects critical points by calculating a composite ERE value based on the structure of biomolecular networks (such as the PPI network), aligning more closely with the basic principles of systems biology. Second, the ERE method only necessitates a reference sample set. The unbalanced number of reference group samples and case group samples (which aligns with realistic scenarios) does not impact the calculation. Based on the ERE method, we successfully identified the critical states in six complex disorders, which were validated according to clinical information or related literature (see Additional file 1: Note S5 for details). Moreover, the edge biomarkers screened from ERE signaling gene pairs can be classified into two categories depending on the outcomes, such as the prognostic survival time of patients, *i.e.*, positive and negative edge biomarkers. According to Kaplan‒Meier survival analysis, we found these edge biomarkers to be statistically significant, including some gene pairs among which there is indeed a biological regulatory relationship that can affect the survival and health of the patient. Moreover, our study shows that gene pairs with differential ERE values clearly indicate a shift in biological states despite non-differential expression of the involved genes. In addition, compared to other methods (including a node-based method and the direct interaction network-based divergence (DIND) method [38]), which focus on individual molecules or local biomolecular direct interaction networks, the ERE method can identify the critical state by exploring differential associations among molecules, providing a systematic and dynamic way to decipher the biological system responding to drug or therapy treatment [39]. Furthermore, as shown in Additional file 1: Fig. S6 and Fig. S20, ERE is effective in identifying the critical points of the simulated data under different noise strengths and groups of edges with highest ERE values, validating the robustness of ERE. To further assess the applied issue of ERE, we have discussed the applicability scenarios and optimal selection of edges for our method. Additionally, we compared the ERE method with pure physical approaches [40, 41] to emphasize its efficiency in identifying critical points in the disease progression. We also analyzed the relationship between ERE and informational entropy as well as thermodynamic entropy [42], to help the scientific understanding of the proposed method and results. Further details can be found in Additional file 1: Note S11. However, the reliance on reference samples and the inability to directly apply the algorithm on the individual sample to be tested constitute limitations of the ERE method.

## Conclusions

In summary, the proposed approach functions as a reliable computing tool with the following advantages. First, in contrast to the common node-based methods, the ERE method is more sensitive to early-warning signals with strong robustness against sample number and noise. Second, the ERE strategy represents a promising way to signal the critical transitions in complex diseases from a gene-pair perspective, which is helpful to track the dynamic changes of cooperative effects on molecular associations. Third, as a model-free computational method, the ERE method does not require model training procedure, differing from conventional classification or machine learning methods requiring massive numbers of samples for supervised or unsupervised learning. Combined with the dynamic prediction method [43] or the statistic-based analysis method [44], the ERE method may help to reveal the dynamic change in molecular associations and networks in a complex biological system near its bifurcation point.

### Supplementary Information

**Additional file 1:** **Table S1.** Traditional gene biomarkers used to detect critical points in each dataset. **Table S2.** The cancer-related signaling pathways enriched by gene pairs with high entropy in critical stage of each dataset. **Table S3.** Genes included in the network of each dataset. **Table S4.** Summary sheets for positive and negative edges of TCGA datasets. **Table S4.1.** Summary sheets for positive and negative edges of COAD. **Table S4.2.** Summary sheets for positive and negative edges of LUAD. **Table S4.3.** Summary sheets for positive and negative edges of THCA and KIRC. **Table S5.** Performance statistics of ERE in various datasets based on the bootstrapping strategy. **Figure S1.** Three states during disease progression. **Figure S2.** Identifying the critical stage for KIRP. **Figure S3.** A schematic illustration for validating the identified critical state. **Figure S4.** Validating the identified critical states of THCA and KIRP. **Figure S5.** The performance of ERE under different sample sizes in numerical simulation. **Figure S6.** Comparison of the performance of ERE under different noise strengths in numerical simulation with other methods. **Figure S7.** Cancer development regulatory mechanisms revealed by ERE signaling gene pairs in KIRC. **Figure S8.** Survival analysis based on positive and negative edge biomarkers for COAD, LUAD, THCA and KIRC. **Figure S9.** A model of 16-nodes network for numerical simulation. **Figure S10.** Probability density functions fit by kernel density estimation based on normal and case samples. **Figure S11.** Comparison of the performances of the ERE method and traditional biomarkers. **Figure S12.** Dynamic evolution of the networks across all stages in each tumor. **Figure S13.** Comparison of the prognosis results based on the identified critical stages by the ERE method and DEGs for THCA. **Figure S14.** Performance of ERE in numerical simulation. **Figure S15.** The performance of ERE on the COAD, LUAD, THCA, and KIRC using a bootstrapping strategy. **Figure S16.** An illustration of the “after-transition state”. **Figure S17.** The three stages transition of interactions between certain genes during the entire disease progression. **Figure S18.** The comparison of the performance of ERE with the standard relative entropy. **Figure S19.** Box plot of ERE values for acute lung injury. **Figure S20.** The performance of ERE under different groups of edges with highest ERE values. **Figure S21.** Comparison of the performance of ERE with that of pure physical approaches. **Note S1.** Details of kernel density estimation.** Note S2.** Details of numerical simulation. **Note S3.** One-sample *t*-test.** Note S4.** Summary for positive and negative edges of TCGA datasets.** Note S5.** Verification for the identified critical state.** Note S6.** ERE gene pairs affect the Rap1 signaling pathway in tumor progression.** Note S7.** KEGG pathway enrichment analysis.** Note S8.** Theoretical basis.** Note S9.** Performance of ERE in numerical simulation.** Note S10.** Details for the expression calculation of DEGs.** Note S11.** Details for the applied issue of ERE. **References S1.**

## Data Availability

COAD, LUAD, THCA, KIRC and KIRP datasets are available from the TCGA database (http://cancergenome.nih.gov). The time-course dataset of acute lung injury (GSE2565) was downloaded from the NCBI GEO database (http://www.ncbi.nlm.nih.gov/geo). All the scripts in this study are available in the GitHub repository: https://github.com/Hongrenhao/ERE.

## Abbreviations

COAD: Colon adenocarcinoma
DEGs: Differentially expressed genes
DIND: Direct interaction network-based divergence
DNB: Dynamic network biomarker
ERE: Edge-based relative entropy
GEO: Gene Expression Omnibus
KDE: Kernel density estimation
KEGG: Kyoto Encyclopedia of Genes and Genomes
KIRC: Kidney renal clear cell carcinoma
KIRP: Kidney renal papillary cell carcinoma
LUAD: Lung adenocarcinoma
non-DEGs: Non-differentially expressed genes
NSCLC: Non–small cell lung cancer
PCC: Pearson correlation coefficient
PPI: Protein–protein interaction
TA samples: Tumor-adjacent samples
TCGA: The Cancer Genome Atlas
THCA: Thyroid adenocarcinoma
