# Supplementary material for: Edge-based relative entropy as a sensitive indicator of critical transitions in biological systems
Source: J Transl Med. 2024 Apr 4;22:333. doi: 10.1186/s12967-024-05145-3 (PMC10996174; doi:10.1186/s12967-024-05145-3)
Supplement: Supplementary file 1 — Additional file 1: Table S1. Traditional gene biomarkers used to detect critical points in each dataset. Table S2. The cancer-related signaling pathways enriched by gene pairs with high entropy in critical stage of each dataset. Table S3. Genes included in the network of each dataset. Table S4. Summary sheets for positive and negative edges of TCGA datasets. Table S4.1. Summary sheets for positive and negative edges of COAD. Table S4.2. Summary sheets for positive and negative edges of LUAD. Table S4.3. Summary sheets for positive and negative edges of THCA and KIRC. Table S5. Performance statistics of ERE in various datasets based on the bootstrapping strategy. Figure S1. Three states during disease progression. Figure S2. Identifying the critical stage for KIRP. Figure S3. A schematic illustration for validating the identified critical state. Figure S4. Validating the identified critical states of THCA and KIRP. Figure S5. The performance of ERE under different sample sizes in numerical simulation. Figure S6. Comparison of the performance of ERE under different noise strengths in numerical simulation with other methods. Figure S7. Cancer development regulatory mechanisms revealed by ERE signaling gene pairs in KIRC. Figure S8. Survival analysis based on positive and negative edge biomarkers for COAD, LUAD, THCA and KIRC. Figure S9. A model of 16-nodes network for numerical simulation. Figure S10. Probability density functions fit by kernel density estimation based on normal and case samples. Figure S11. Comparison of the performances of the ERE method and traditional biomarkers. Figure S12. Dynamic evolution of the networks across all stages in each tumor. Figure S13. Comparison of the prognosis results based on the identified critical stages by the ERE method and DEGs for THCA. Figure S14. Performance of ERE in numerical simulation. Figure S15. The performance of ERE on the COAD, LUAD, THCA, and KIRC using a bootstrapping strategy. Figure S16. An illustration of [file 12967_2024_5145_MOESM1_ESM.docx]

**Additional file 1**

**“Edge-based relative entropy as a sensitive indicator of critical transitions in biological systems”**

Renhao Hong, Yuyan Tong, Huisheng Liu, Pei Chen, Rui Liu

Contents

[**Section S1. The supplementary tables** 3](#_Toc161779070)

[Table S1. Traditional gene biomarkers used to detect critical points in each dataset 3](#_Toc161779071)

[Table S2. The cancer-related signaling pathways enriched by gene pairs with high entropy in critical stage of each dataset. 3](#_Toc161779072)

[Table S3. Genes included in the network of each dataset. 4](#_Toc161779073)

[Table S4. Summary sheets for positive and negative edges of TCGA datasets 6](#_Toc161779074)

[Table S4.1 Summary sheets for positive and negative edges of COAD 6](#_Toc161779075)

[Table S4.2 Summary sheets for positive and negative edges of LUAD 7](#_Toc161779076)

[Table S4.3 Summary sheets for positive and negative edges of THCA and KIRC 8](#_Toc161779077)

[Table S5. Performance statistics of ERE in various datasets based on the bootstrapping strategy 8](#_Toc161779078)

[**Section S2. The supplementary figures** 9](#_Toc161779079)

[Fig. S1. Three states during disease progression 9](#_Toc161779080)

[Fig. S2. Identifying the critical stage for KIRP 10](#_Toc161779081)

[Fig. S3. A schematic illustration for validating the identified critical state 10](#_Toc161779082)

[Fig. S4. Validating the identified critical states of THCA and KIRP 11](#_Toc161779083)

[Fig. S5. The performance of ERE under different sample sizes in numerical simulation 11](#_Toc161779084)

[Fig. S6. Comparison of the performance of ERE under different noise strengths in numerical simulation with other methods 12](#_Toc161779085)

[Fig. S7. Cancer development regulatory mechanisms revealed by ERE signaling gene pairs in KIRC 13](#_Toc161779086)

[Fig. S8. Survival analysis based on positive and negative edge biomarkers for COAD, LUAD, THCA and KIRC 14](#_Toc161779087)

[Fig. S9. A model of 16-nodes network for numerical simulation 15](#_Toc161779088)

[Fig. S10. Probability density functions fit by kernel density estimation based on normal and case samples 16](#_Toc161779089)

[Fig. S11. Comparison of the performances of the ERE method and traditional biomarkers 17](#_Toc161779090)

[Fig. S12. Dynamic evolution of the networks across all stages in each tumor 18](#_Toc161779091)

[Fig. S13. Comparison of the prognosis results based on the identified critical stages by the ERE method and DEGs for THCA 19](#_Toc161779092)

[Fig. S14. Performance of ERE in numerical simulation 20](#_Toc161779093)

[Fig. S15. The performance of ERE on the COAD, LUAD, THCA, and KIRC using a bootstrapping strategy 21](#_Toc161779094)

[Fig. S16. An illustration of the “after-transition state” 22](#_Toc161779095)

[Fig. S17. The three stages transition of interactions between certain genes during the entire disease progression 23](#_Toc161779096)

[Fig. S18. The comparison of the performance of ERE with the standard relative entropy 24](#_Toc161779097)

[Fig. S19. Box plot of ERE values for acute lung injury 25](#_Toc161779098)

[Fig. S20. The performance of ERE under different groups of edges with highest ERE values 26](#_Toc161779099)

[Fig. S21. Comparison of the performance of ERE with that of pure physical approaches 27](#_Toc161779100)

[**Section S3. The Supplementary Notes** 27](#_Toc161779101)

[Supplementary Note S1. Details of kernel density estimation 27](#_Toc161779102)

[Supplementary Note S2. Details of numerical simulation 28](#_Toc161779103)

[Supplementary Note S3. One-sample *t*-test 31](#_Toc161779104)

[Supplementary Note S4. Summary for positive and negative edges of TCGA datasets 32](#_Toc161779105)

[Supplementary Note S5. Verification for the identified critical state 32](#_Toc161779106)

[Supplementary Note S6. ERE gene pairs affect the Rap1 signaling pathway in tumor progression 34](#_Toc161779107)

[Supplementary Note S7. KEGG pathway enrichment analysis 34](#_Toc161779108)

[Supplementary Note S8. Theoretical basis 35](#_Toc161779109)

[Supplementary Note S9. Performance of ERE in numerical simulation 37](#_Toc161779110)

[Supplementary Note S10. Details for the expression calculation of DEGs 38](#_Toc161779111)

[Supplementary Note S11. Details for the applied issue of ERE 38](#_Toc161779112)

[**Supplementary references** 42](#_Toc161779113)

# Section S1. The supplementary tables

## Table S1. Traditional gene biomarkers used to detect critical points in each dataset

| Datasets | COAD | LUAD | THCA | KIRC |
| --- | --- | --- | --- | --- |
| Biomarkers | *YY1AP1* | *BTG2* | *SYT12* | *STEAP3* |
|  |  |  |  | *IBSP* |
|  |  |  |  | *AQP9* |
| Relation with cancer | *YY1AP1* may serves as a prognostic biomarker in colon adenocarcinoma [1]. | *BTG2* serves as a potential prognostic marker and correlates with immune infiltration in lung adenocarcinoma [2]. | *SYT12* may contribute to predicting THCA outcomes as a novel biomarker [3]. | *STEAP3*, *IBSP* and *AQP9* exert important effects on prognostic significance analysis for renal cell carcinoma [4]. |

## Table S2. The cancer-related signaling pathways enriched by gene pairs with high entropy in critical stage of each dataset.

| Gene pairs | Enriched pathways | Datasets |
| --- | --- | --- |
| BDNF-NTRK2, CSF3-IL6, CXCL12-LPAR3, CXCL8-IL10, CXCL8-IL1B, CXCL8-IL6, CXCL8-TLR2, CXCL8-TLR4, FGF2-SOX2, FGF8-SOX2, FGF9-FLT1, FGF9-KDR, FST-INHBB, FZD10-WNT16, FZD10-WNT7A, IFNG-STAT1, IL12A-IL12RB1, IL1B-IL6, IL6-JAK2, IL6-LEP, IL6-OSM, IL6-STAT1, NGFR-NTRK2, PIAS2-STAT1, SOX2-STAT3 | TGF-beta signaling pathway, JAK-STAT signaling pathway, HIF-1 signaling pathway, Hippo signaling pathway, PI3K-Akt signaling pathway, NF-kappa B signaling pathway | COAD |
| BTRC-PRLR, CACNA1D-CACNG8, CCNE1-CDK2, DKK1-FZD1, DKK1-MYC, DKK1-WNT1, EGFR-EREG, EGFR-MYC, EGFR-RAC1, FLT4-VEGFC, FOSL1-MYC, FZD1-WNT9B, GAPDH-MYC, GNB1-GNG4, GNB2-GNG4, GNB3-GNG4, GNB4-GNG4, GNB5-GNG4, GNG11-GNG4, LDHA-MYC, LGR4-RSPO3, LGR5-RSPO1, LGR5-RSPO2, MAPK7-RPS6KA2, PRKCG-RHOA, PRL-PRLR, WIF1-WNT11 | MAPK signaling pathway, HIF-1 signaling pathway,  Wnt signaling pathway,  PI3K-Akt signaling pathway | LUAD |
| ADCY1-GNAS, ADCY1-PRKACB, CNTF-CNTFR, CNTFR-LIFR, CYP1A2-GSTM1, EPHX1-GSTM1, GSTA1-GSTA2, GSTM1-GSTM2, GSTM1-GSTM3, GSTM1-GSTO1, LDHC-PDHA1, PPARGC1A-SLC2A4 | Chemical carcinogenesis-receptor activation, JAK-STAT signaling pathway, AMPK signaling pathway, HIF-1 signaling pathway | THCA |
| BMP4-NOG, BMP5-NOG, BMP6-NOG, BMP7-NOG, DLG1-GRIN2A, DLG2-GRIN2A, EPO-EPOR, EPO-JAK2, GDF6-NOG, GRIN2A-GRIN2B, PPP2R2C-PPP2R5A | PI3K-Akt signaling pathway, Hippo signaling pathway, Rap1 signaling pathway, TGF-beta signaling pathway | KIRC |

## Table S3. Genes included in the network of each dataset.

| Dataset | Critical stage (time point) | Genes involved in networks |
| --- | --- | --- |
| COAD | IIB | WDHD1, SHH, SLCO1B1, POU5F1, RPL38, RGS4, BSG, EPHA4, TUBA4A, TFR2, RTN4R, CDX2, DLX2, RPS10-NUDT3, RHOA, MYH3, FZD1, DNAJB14, PAK2, GNAQ, TP53, RPL7A, CTNNB1, NTS, EEF1A1, RPS16, RPL10, ARHGEF15, TPM4, FANCL, ATRIP, DRD2, NMB, FZD8, RPL31, CYP1A1, FANCB, TNNI3, PMCH, EPOR, EIF3F, NANOGP8, RPS3, IL12RB1, BAG4, IL2, SGTA, LEF1, TNNI1, IFNG, SIX3, FOXA1, EIF4E, WNT16, MAPK8, IGFBP6, SOCS1, TNNC1, TTR, CCL8, ZWILCH, RPL7L1, UGT2B7, STAT1, MRPL4, RPS5 LPAR6, ALB, CXCL2, IGFBP3, LRP6, IDE, SMAD3, RPL6, SOCS2, RPL11, TF, GATA6, PLA2G2A, RPSAP58, SF1, HSPE1, PYY, SST, MRAP, FZD5, SERPINE1, GHR, CXCL10, BMP4, IL12RB2, CXCL13, RPL3L, IRF8, IL1B, BCL2, RPS26, SERPINC1, GPC3, RPS15, MEF2C, ALOX15B, RPL35A, MRPL2, RTN4, GRPR, PF4, APOA4, RPL26, SMAD4, C3, F13A1, PPARGC1A, TPM3, CCL2, CCKAR, NOG, IL17A, PCSK1, TIPIN, GANAB, RPL17-C18orf32, CYP2E1, FZD3, ZNF408, RPL10L, EIF5, ATF2, ZSCAN10, WNT8A, IL10, F7, IGFBP5, OSM, SHC1, RPS15A, GSTT2B, FASLG, RPL13, BCAR1, FANCM, SDC1, PIAS2, RPS3A, HP, WNT1, CXCL8, MRPS5, TNNI2, MCHR1, BRF1, BMP5, FOS, TBX21, HSP90B1, PIAS1, RPL22L1, CCR2, KLK4, ACKR1, POLR1D, NFKBIA, RPL27A, JUND, MYF5, INS, KRBA2, HEY1, SERPINB5, SMAD2, RPS17, RPL32, DKK1, NPY1R, STAT5A, NPS, CCL27, POU4F1, HSPBP1, GIP, BUB1, RPL13A, KLF4, TUBB4A, HPSE, FGF2, IGFBP1, BMP2, NPSR1, TCF7L2, EIF3I, TAC1, RPS4Y1, SSTR5, SOX2, POLR1E, RPL37A, CP, LRP2, MLN, EN1, RPS18, NME1, RPL35, APOA2, FANCG, EDN1, PRKCSH, RPL39, SPDEF, SMC1B, MEF2D, SSB, RPL36A, IGF1, AXIN1, RPL27, RMI1, STAT4, FZD6, EIF2S3, AR, DLX1, MYC, TNNC2, SLC9A3, RPL5, IMP3, POLRMT, CXCR1, RPL37, FANCC, NTSR2, WIF1, RPL39L, INCENP, APOA1, RPL18, FZD10, TIMP1, CCL21, GSTM1, IL6ST, WNT8B, FAS, CXCR2, RAC1, RPL17, ARNTL2, SNTA1, RPL3, RPL21, ZFPM2, SLCO1A2, F5, JAK2, SOCS3, EIF1AX, LRP5, RPS9, PLA2G7, RPL24, CCL5, TFPI, MAPK12, MLNR, WNT5B, GHSR, CITED1, PRL, CD36, MTTP, BNIP3, RPS25, LRP1, GNB2, TRH, EIF4B, NMU, TTN, HSPA9, PLA2G4A, RPLP2, KIF2C, RPL23A, APOE, HEPH, WLS, LINGO1, RPS19, DVL2, RPL15, KNG1, HSF1, CUBN, ABCC3, RPL34, SPDL1, PLA2G5, CASR, HRK, PTPN11, RPS12, WNT3A, CNPY3, NTF4, CXCL9, CCR1, PNOC, CXCL5, CANX, FANCD2, MAP2K4, CAV1, LPAR5, MIS12, KREMEN1, GPC1, FOXD3, HS3ST5, HEY2, RPS23, RPS11, CCL20, PTPN13, PTH, SRF, TYK2, CLSPN, RPL22, VWF, F2RL3, NPY, WNT5A, AVP, IGF2BP2, CCKBR, RBBP8, HSPD1, RSRC1, CXCL6, EIF2S1, SHC3, PENK, RPL26L1, CCR9, BDNF, NFATC4, MAPK3, GIPC1, IL18, PON1, RPL14, RPL18A, HSPA8, CSF3R, CDC42, PLK4, LPAR3, PTGS2, SHC4, GNAI3, RPS24, RPL10A, AKT2, RPL36AL, DVL1, RPS13, ZFYVE16, BAG2, EFNA5, RPSA, RPLP0, RPL19, NTRK2, RPS14, NKX3-1, HSP90AA1, RPL28, TBCD, CAV2, EIF4A2, STAT3, TOP3A, SALL4, SLC40A1, GSTM2, TP53AIP1, SMAD5, HIF1A, MYH7, HAND2, WDR31, TLR4, DUSP6, DNAJB2, LRRK2, ELANE, EN2, LIN28A, OLR1, NCF2, CCR7, FANCE, CDK2, WNT7B, MRPL33, IL6, ESPL1, NDST1, GCG, CSF2, ICAM1, CCL19, FZD2, RACK1, RPL8, BCL2L1, TLR2, STUB1, LMX1A, FANCF, IL1A, LPL, IGFALS, CCK, CSF2RB, RPS21, FAU, MAG, RPS20, RPL23, FGF8, RPS7, EIF1AY, RPS29, TNNT1, CETP, CCL4, MRPL1, DNAJB1, CCL28, IL12A, JUN, CPB2, SHC2, BMP7, RPS27, SIRPG, EIF3G, MRPL12, CYP2B6, ZIC3, SH2B1, UBC, NTF3, MYL3, CNTF, RELA, CCR5, RPL30, MAP2K6, MYL2, SFRP5, WNT4, NR1I2, PAX6, CCL25, CXCL11, TNNT3, ABCA1, GJA5, APOL1, WNT7A, GPC5, RPL36, SAR1B, F11, IL1R1, CXCR3, MYF6, SRC, CSF3, SSTR1, RPL7, RPS8, TPM1, DUSP16, GRP, CIITA, POU2F1, GRM3, NGFR, PLCG1, IGF2, RPA2, MAPKAPK3, CXCL12, NGF, CFD, IGFBP4, PPBP, OXT, EEF2, KREMEN2, RPL12, DUSP1, KNL1, CREB1, TDGF1P3, NKX2-5, GBP1, EOMES, RPL4, GNG3, RPS4X, DDX17, NDC80, MOGS, ZMYND19, MMP1, RPS2, TIMM44, ESR1, ALOX12, SYCP2, CCR10, POLR3D, RPS6, PTGES3, F10, IL18R1, APOB, RPS27L, RPS28, GATA4, ANKRA2, TFRC, IRF1, TBX5, RPL29, RPL9, BARD1, RRP1, LEP, F2 |
| LUAD | IIIB | MIS12, RSRC1, LBP, NPR1, FN1, CYP2D6, BAMBI, IAPP, TF, RPS28, CDT1, RPL18, WNT9B, TUFM, CPS1, ACADSB, PDHA2, GNB4, F13A1, TP53, ACADM, EEF1B2, FGA, ADCY1, ADCY2, MUC16, UGT1A6, VEGFA, BTRC, EGFR, RPL15, DLK1, CDC6, MYC, GK, RPS4Y1, CYP2B6, APOA1, MUC21, RPL26L1, ACSS1, MUC3A, UBC, RPL6, EREG, RPL12, APOA5, EIF3I, RPL10L, HNF1B, ADH1C, FST, GALNT12, IGF1, MUC6, MRPS5, FAU, F2, RPS14, HNF4A, RPSA, RPL23, RPL39L, BMP4, RAC1, RPS15A, GAPDH, FGG, FOXA2, ADH6, NEUROD1, RPL13A, RPS23, CYP2C8, GNG4, RPS5, RPL9, RPS3, DRP2, ADCY5, FGF10, RPL39, HPX, APOA2, C8A, PRKACA, HMGCS2, CYP3A5, ALDH3A1, RPL27, F13B, LRP2, RPS12, F5, HIBCH, RPL27A, DPYSL3, ACAT2, CCNE1, MUC5AC, MUC5B, TRPM2, RPS16, MUC7, PRKAR2A, MAFA, FOSL1, SOCS3, ALDH3A2, PDHB, ITGB3, RPS18, RPL10, GNAI3, CYP1B1, CYP1A1, EEF2, HADH, GNG11, KRBA2, RPL36AL, RPS11, RPL14, FGB, GNB3, PDE11A, DKK1, PVALB, ACLY, RPS20, LPL, FOXO1, PRLR, RPL17, RPS27, GNB1, HMGA1, CASR, CYP3A4, CDKN3, INS, RPL7L1, EIF3F, GSTM1, OGDH, EIF1AY, APP, C8G, LDHA, NIFK, UGT2B7, RPL4, RPS21, LCAT, RGS7, BMPR1B, GNB5, WDR31, PDE7B, C8B, MUC2, RPL28, PDHA1, PDX1, ACVR2A, F2RL2, PF4, RPS8, EIF2S3, RPLP0, BMP7, AOX1, APOB, CDK2, RPL36A, ITGA2B, PRL, RPS24, SKP2, RPL11, SERPINC1, RB1, RPL7, MUC12, GNAI1, RPS19, PPARA, GNB2, MUC13, WNT1, DPYSL4, RACK1, RGS7BP, MUC17, RPL21, RPS3A, PEMT, HNF1A, EIF3G, FZD1, RPS15, ALB |
| THCA | II | EN2, PTPRO, TRPC5, OXSM, TST, SSTR3, POMC, PPY, DBT, LMX1B, TACR3, KITLG, GRK2, APOL1, CAD, ACOX3, TFF1, PAX5, FOS, PLTP, GRB10, GNRH1, SRD5A1, ACACA, LIPE, HADH, BMP2, EDN1, HMGCS2, PDHB, FABP4, DLST, PIK3CG, SST, AR, IDH3A, PHYH, FLT4, UQCRHL, ABCA1, NOG, VEGFA, INS, GCSH, SET, MPST, STAT5B, UBC, SCARB1, GRM8, CPS1, BMP7, COX6C, BCAT2, PKM, UMPS, MAPK8IP1, MYC, FH, MLST8, LBP, DOK1, EDNRA, POLB, LEP, ESRRG, ACAT1, SDHA, NDUFS3, NPY, SUCLA2 TRIB3, DMGDH, PDPR, AGXT, VDAC1, IDH1, JAK2, SOCS1, CETP, APOB, MDH1, UQCRQ, HSD3B2, SLC25A20, MATK, UQCR10, BCL2, RUVBL1, AGT, SGK1, TRAM1, CEBPA, RAPGEF3, ACAT2, NOS3, CDK5, TF, LRP2, MSX1, HSD17B3, CPT2, TBC1D4, ACE2, NPY5R, SHMT1, PC, LDHAL6A, HPR, ME3, MYB, NCOA6, LIG4, NDUFAB1, NR0B2, PRKDC, FZD3, COX4I1, ENDOG, ASNS, SLC4A8, COX7C, KCNJ8, PCK1, ACSS2, LDHC, IYD, LIG1, SHC1, PGF, APOE, CARTPT, ROCK2, TACR2, MIS12, OGDHL, MAPT, MAPK12, CRKL, CRHR2, IGF1R, PPARG, COX4I2, FOXA1, HMGB1P1, COX6B1, LCK, SSTR2, GRB14, ARRB1, HSPB1, ABCG1, AIFM1, PON1, SDHC, ANK2, BMP4, AHI1, EDN3, GRAP, OGG1, SH3GL2, PPARGC1A, DPYSL4, IDH3B, APOA5, OXCT2, PPIF, KCNJ1, ADIPOQ, STUB1, DNTT, DNM1, HMGB2, PLIN1, STAT3, CS, ME2, CYP17A1, ABCC8, GNRH2, FOXA2, CBLB, GNAQ, EDNRB, STAT5A, NDUFA4, ESRRA, BIN1, PPARGC1B, HADHB, HS3ST6, ACO1, UQCRFS1, PTPN11, APP, UCN, AVPR1A, RAG1, PDCD6IP, SLC25A4, MTTP, XRCC1, POLD1, GRK5, NKX6-2, WNT7A, AKT2, ANP32A, BCKDHB, ACAA1, UQCRC2, NRIP1, KISS1, HMGCR, GBX2, CFTR, FGF9, HAP1, GLI2, WNT3A, SHMT2, AGTR1, HSD17B2, TEC, PROK2, DHODH, APOC3, CDON, WIF1, PIK3CD, CDK6, DLD, CARNS1, CTNNB1, VIP, OGDH, RGS16, COX6A1, SOSTDC1, NME1, KPNA1, NR4A1, CBL, XRCC6, COX5A, FZD10, ALB, GNAI1, GOT2, GPC5, PRKAA2, NPY1R, WNT4, NKX6-1, SUCLG1, VLDLR, SNCA, SHH, STAP1, ESR1, RAG2, TAC3, APOA2, IRS4, PHLPP1, MARK1, NR4A2, FEN1, LDHB, LCAT, CYCS, AGXT2, PMCH, FKBP4, KIT, PCNA, TPO, TAC1, KNG1, EGFR, LYN, ME1, CNR1, FKBP1A, AKT1, CYC1, CRH, LPL, ISL1, ACADM, HSD3B1, ITPR1, PHLPP2, PDHA1, GLDC, ACADS, ACLY, CD36, YY1, SARDH, PRKCA, PPARA, UQCRC1, SUCLG2, ACO2, KCNJ11, PDK4, PVALB, SH2B3, SH3KBP1, MPL, VDAC3, LRP8, MTHFD2, IHH, ACACB, RAPGEF4, GZMA, GOT1, NDUFB10, ABCA7, XRCC5, LEF1, NDUFS2, SLC2A4, APOA1, SFRP1, RGS14, KLK11, HTT, ALDH1L1, PMPCB, HP, SLC9A3R1, HSD17B6, NTHL1, SDHD, ACADSB, MDH2, TFCP2, IDH2, ADCY9, FZD4 |
| KIRC | II | HMCN1, SLC9A3R2, CCL5, CCL16, CXCL1, RASGRF2, PTK2, DLG1, HBG2, CST3, SLA2, B2M, DNM2, PPP2R2C, NR4A2, PPP2R1B, PTPN22, PAX6, FGG, TP53, PPBP, CXCR1, TNFRSF9, CUBN, RAD52, GSN, CCR2, CD3E, CYSLTR2, CAMK2D, NOP9, TTN, CCL20, TFF1, DSCAM, ERCC8, POU5F1, TIMM50, CXCL10, ARG2, SAA2, IL12RB2, NCAM1, PFDN2, CCR4, NKX2-2, CD40LG, CHP1, OLIG2, CD47, PPP2R5C, SCGB1A1, NEUROG3, AIFM1, BMP4, CD84, MYPN, LYZ, CCT8, EDN1, KIT, SLIT2, CCL21, IRF1, BMP5, SIRPG, FOXA2, BMPER, ACTA1, TRAF2, TRDN, MMP1, CD8B, FGA, BMPR1B, PPP2R5A, CD22, CXCL11, GDF5, HEPH, OMG, CD27, BMP10, AHSP, PFDN4, TTR, COX5A, CCL19, SLAMF6, PLA2G4A, NKX6-1, SALL4, RYR2, ODC1, PFDN5, IL12B, PTPRC, CXCL5, F13B, DEGS2, LRP2, EGFR, CCR5, LCK, CXCL6, FOXA3, PAK2, SLK, CD79A, ROCK2, CCT4, EPO, FOXA1, F13A1, TF, CCL2, AKAP9, HMGCS2, HAND1, CCR3, F2, IL6, CXCL2, TRAM1, GRIN2A, STAT1, GATA4, SMAD4, CD19, PIGR, WNT7A, CCR6, JAK2, DCC, SPHK1, UQCRC2, CD80, IAPP, HIF1A, GP1BA, UNC5A, APP, HLA-C, GRIN2B, PIGA, TBX21, IGFBP1, SHH, CCT2, CCT7, APOA1, PTGS1, APOA4, F7, CP, HBA2, FOXP3, DNM1, CCT5, CASQ2, CXCR2, EOMES, SGK1, ICOS, NTN1, TUBA3D, BMP7, ICAM1, OTC, PPP2R1A, SIX3, TUBA3E, IL2, TNNT1, NANOGP8, IFNG, SRC, TBXAS1, CCL11, CD3D, SOX10, GRIN1, NEB, F11, CYCS, TBX5, CCNH, SH2D2A, HSPG2, CERS1, SLAMF1, BMP6, STAT4, AMPH, PSAP, CD244, SH3GL2, TNNI3, CXCL8, CXCL9, MIS12, CBL, DAPP1, CXCL13, GDF6, SLC9A3, F10, CD70, CCR8, ST8SIA4, PPP4C, NDUFAB1, HADHB, HBD, GRIN3A, CCL17, PRKACG, RBP4, ACAT1, MMP7, GC, F2RL1, CYSLTR1, UNC5D, SH2D1A, FGB, TCP1, HLA-B, MAG, LMX1A, RAB3B, NOG, ZAP70, UBC, RTN4, HNF4A, PTK2B, ITGAM, PROC, NKX2-5, PTGIS, ITGB3, IL12RB1, CD69, MAL, ST8SIA2, PLA2G5, SLC40A1, PPP2R3A, FOSL1, SRF, DLG2, TNFSF9, MNAT1, CXCR3, FPR1, PDCD6IP, CCT6A, UQCRFS1, SOX2, DES |
| Lung injury | 8h | Bms1, Prkcq, Utp3, Brca1, Nbn, Eif3c, Cd3g, Cd3e, Lgals3, Il2ra, Arid1a, Ctnnb1, Cry2, Lmnb1, Mapk3, Rps11, Nol5, Dvl3, Nanog, Magi2, Anapc1, Top2a, Gnb2l1, Rps3, Eif2s1, Ensmusg00000059776, Rpl21, Wdr31, Rpl35, Csnk1e, Ror1, Dvl1, Rpl22, Eif4a2, Fas, Lmna, Rpl39l, Pitx2, Brd4, Emd, Rplp2, Pias4, Rps15a, Rpl14, Sgol1, Eif2s2, Chek1, Rpl11, Ube2f, Eif3k, Cdc37, Mki67ip, Rpl6, Bysl, Magi3, Rpl12, Rpl19, Csnk1a1, Casp3, Noc4l, Ube2i, Cdc45l, Hfe, Pros1, Lck, Ggcx, Rpl3l, Cdc27, Mki67, Tlr2, Cd4, Hspb2, Cflar, Vkorc1, Rps25, Rps14, Ngdn, Eif1, Tradd, Klrd1, Cd3d, Fzr1, Zap70, Rpl15, Daxx, Ripk1, Rb1, Pih1d1, Gins4, Eef2, Eif3h, Irs1, Rpl26, Clock, Abl1, Chd7, Rps9, Brd7, Lmnb2, Prc1, Hist2h3c2, Eif1b, Sumo3, Cry1, Rpl18, Tjp1, Setd8, Dcun1d1, Stat3, Myc, Rpsa, Rps19, Foxo1, Ube2v2, Smarca4, Utp20, Wdr36, Eif3d, Mdm2, Pttg1, Tbl1x, Cdc2a, Hsp90aa1, Rplp1, Wnt3a, Prkca, Prmt5, Ppp5c, Rps18, Arrb1, Rps4x, Per1, Cd8a, Smarcc1, Anapc13, Rpl39, Ubc, Rps17, Smarcd3, Ptprm, Rps27l, Eif3a, Pou5f1, Ctnna1, Atm, Mtbp, Tap1, Smarcb1, Yap1, Il2rb, Tapbp, Cish, Rpl22l1, H2-k1, Rad50, Ensmusg00000050299, Brwd1, Mad2l1, Dusp9, Fzd2, Ensmusg00000058905, Sall4, F7, H2-t18, Pbrm1, Jup, Trp53, Jun, Tnfaip3, Rpl7, Eif3g, Sox17, Rpl8, Kptn, Cd44, Wdr46, Itk, Anapc11, Aurka, Ltv1, Cd247, Prdx2, Cd8b1, Spata13, Rps6, Uba3, Sox2, Ar, Cd28, Eif4a1, Rps24, Rps2, Ptpn13, Ensmusg00000059775, Apc, Arntl, F2, Cdh15, Rplp0, Trp63, Proc, Pik3r1, Rpl4, Rps15, Sag, Mertk, Nedd9, H2-eb1, Mta2, Fkbp4, Park7, Imp3, Timeless, Mcm10, Ube2c, Rps5, Anapc4, Sumo1, Rps27, Fbl, Il2, Cdk5, Unc84b, B2m, Atpbd4, Ndc80, Eif3j, Hdac2 |

## Table S4. Summary sheets for positive and negative edges of TCGA datasets

**Table S4.1** Summary sheets for positive and negative edges of COAD

| COAD | | |
| --- | --- | --- |
| Edges | **P-value** | **Positive/Negative** |
| DEFA6-DEFB1 | 0.027 | Positive |
| LINGO1-RTN4 | 0.0016 | Negative |
| CERS3-SPHK1 | 0.0091 | Negative |
| LINGO1-NGFR | 0.011 | Negative |
| ELOVL2-FADS1 | 0.019 | Negative |
| TNNC1-TNNT1 | 0.036 | Negative |
| GSTM1-GSTT2B | 0.042 | Negative |

**Table S4.2** Summary sheets for positive and negative edges of LUAD

| LUAD | | | | | |
| --- | --- | --- | --- | --- | --- |
| Edges | **P-value** | **Positive/Negative** | **Edges** | **P-value** | **Positive/Negative** |
| ADH1C-GSTM1 | 0.0068 | Positive | NEUROD1-PDX1 | 0.014 | Negative |
| GPC5-HS3ST6 | 0.015 | Positive | CCL20-CXCL10 | 0.017 | Negative |
| WIF1-WNT11 | 0.019 | Positive | EGFR-RAC1 | 0.019 | Negative |
| DKK1-FZD1 | <0.0001 | Negative | LGR4-RSPO3 | 0.019 | Negative |
| DKK1-MYC | <0.0001 | Negative | PDHB-PDX1 | 0.019 | Negative |
| GAPDH-MYC | <0.0001 | Negative | PDHA2-PDX1 | 0.021 | Negative |
| BCAN-MMP10 | 6.00E-04 | Negative | ACLY-PDX1 | 0.022 | Negative |
| F2-F5 | 0.0012 | Negative | TNNI3-TNNT1 | 0.022 | Negative |
| HMGA1-MYC | 0.0014 | Negative | GK-PDX1 | 0.028 | Negative |
| OGDH-PDX1 | 0.0023 | Negative | GNB1-GNG4 | 0.028 | Negative |
| HNF1B-PDX1 | 0.0024 | Negative | FOSL1-MYC | 0.029 | Negative |
| IAPP-PDX1 | 0.0025 | Negative | F5-PF4 | 0.03 | Negative |
| SIRT1-SOD2 | 0.0031 | Negative | ACVR2A-BMP7 | 0.035 | Negative |
| AADAT-KYNU | 0.0034 | Negative | FOXO1-PDX1 | 0.039 | Negative |
| DKK1-WNT1 | 0.004 | Negative | GATA3-MYB | 0.039 | Negative |
| MAFA-PDX1 | 0.0049 | Negative | HNF1A-PDX1 | 0.041 | Negative |
| INS-PDX1 | 0.0071 | Negative | FOXA2-PDX1 | 0.043 | Negative |
| UPK1A-UPK2 | 0.0075 | Negative | EIF2S3-RPS4Y1 | 0.046 | Negative |
| EGFR-MYC | 0.012 | Negative | ALB-HPX | 0.047 | Negative |
| PDHA1-PDX1 | 0.013 | Negative | IDO1-KYNU | 0.047 | Negative |

**Table S4.3** Summary sheets for positive and negative edges of THCA and KIRC

| THCA | | | KIRC | | | |  |
| --- | --- | --- | --- | --- | --- | --- | --- |
| Edges | **P-value** | **Positive/Negative** | | **Edges** | **P-value** | **Positive/Negative** | |
| LMX1B-NR4A2 | <0.0001 | Negative | | F2-TF | 0.0027 | Negative | |
| MPL-TPO | 0.0085 | Negative | | F7-TF | 0.0028 | Negative | |
| KCNA1-KCNA2 | 0.0089 | Negative | | C1QA-CRP | 0.0039 | Negative | |
| DGKI-PLCD4 | 0.011 | Negative | | BMP7-NOG | 0.0073 | Negative | |
| NEB-TPM3 | 0.021 | Negative | | HIF1A-TF | 0.02 | Negative | |
| RAG1-RAG2 | 0.049 | Negative | | TF-TFR2 | 0.032 | Negative | |

## Table S5. Performance statistics of ERE in various datasets based on the bootstrapping strategy

| COAD | | |
| --- | --- | --- |
| stage | count | percent |
| I | 0 | 0.00% |
| IIA | 0 | 0.00% |
| IIB | 499 | 99.80% |
| IIIA | 1 | 0.20% |
| IIIB | 0 | 0.00% |
| IIIC | 0 | 0.00% |
| IV | 0 | 0.00% |
| LUAD | | |
| stage | count | percent |
| I | 0 | 0.00% |
| IIA | 0 | 0.00% |
| IIB | 0 | 0.00% |
| IIIA | 0 | 0.00% |
| IIIB | 500 | 100.00% |
| IV | 0 | 0.00% |
| THCA | | |
| stage | count | percent |
| I | 0 | 0.00% |
| II | 481 | 96.20% |
| III | 1 | 0.20% |
| IV | 18 | 3.60% |
| KIRC | | |
| stage | count | percent |
| I | 0 | 0.00% |
| II | 493 | 98.60% |
| III | 7 | 1.40% |

# Section S2. The supplementary figures

## Fig. S1. Three states during disease progression


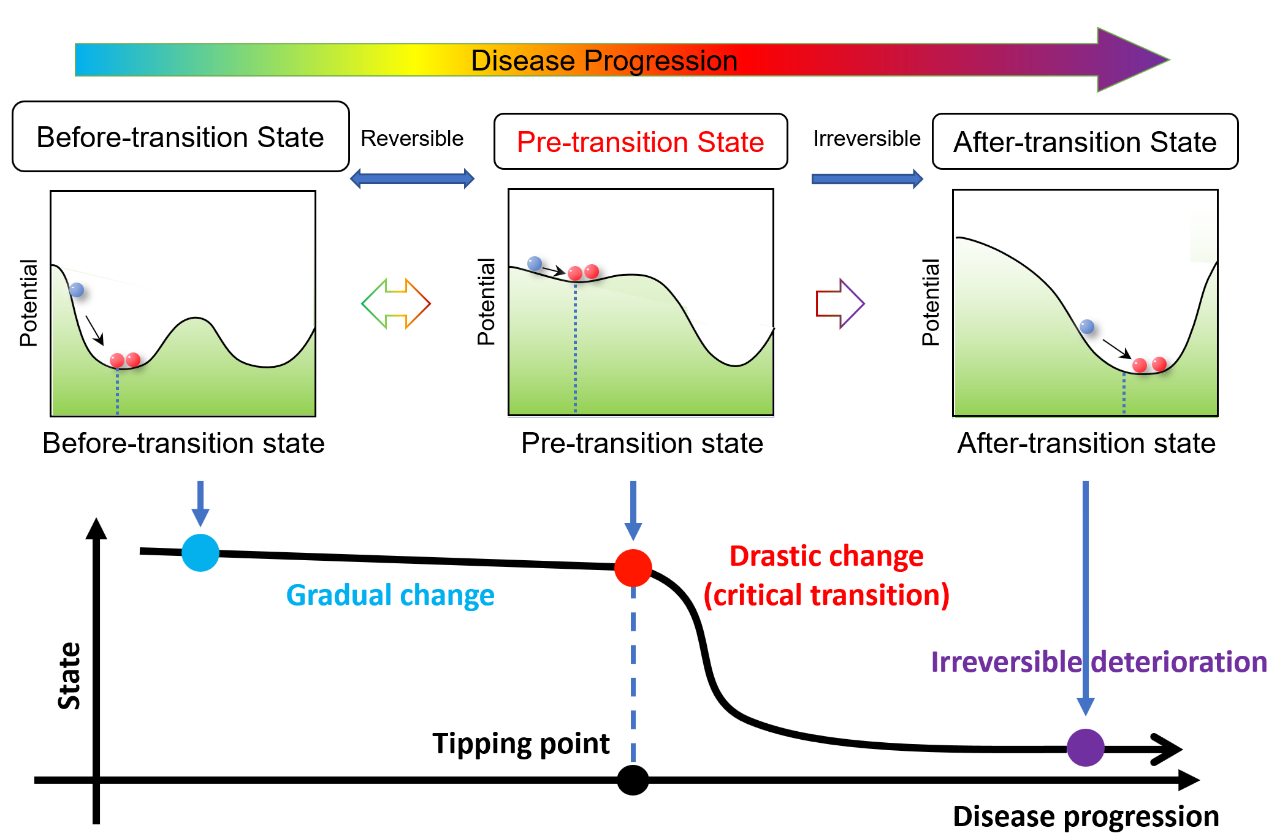


**Figure S1.** **Three states during disease progression.** The before-transition state is a steady or stable state with strong resilience for small perturbations, representing a relatively healthy stage. The pre-transition/critical state is defined as the limit of the before-transition state but with a lower resilience from small perturbations. Such a pre-transition state is the critical stage during the disease progression. When the system is at the pre-transition state, timely and proper medical intervention can bring the system back to the before-transition state. The after-transition state is another stable state with strong resilience, where the system turns into a severe deterioration stage and thus it is generally difficult to return to the before-transition state even by the intensive medical treatment.

## Fig. S2. Identifying the critical stage for KIRP


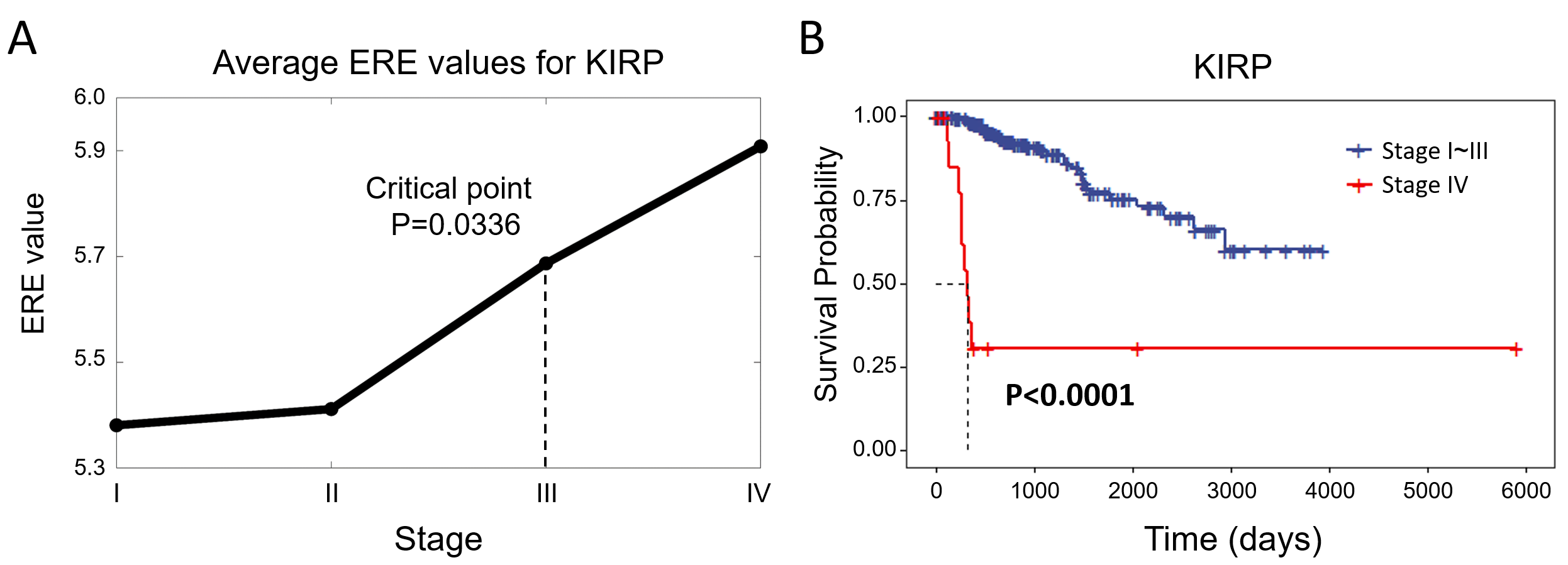


**Figure S2.** **Identifying the critical stage for KIRP.** (A) The ERE curve calculated from gene expression data of KIRP. (B) Survival curves for patients of KIRP obtained before and after the critical state.

## Fig. S3. A schematic illustration for validating the identified critical state

**
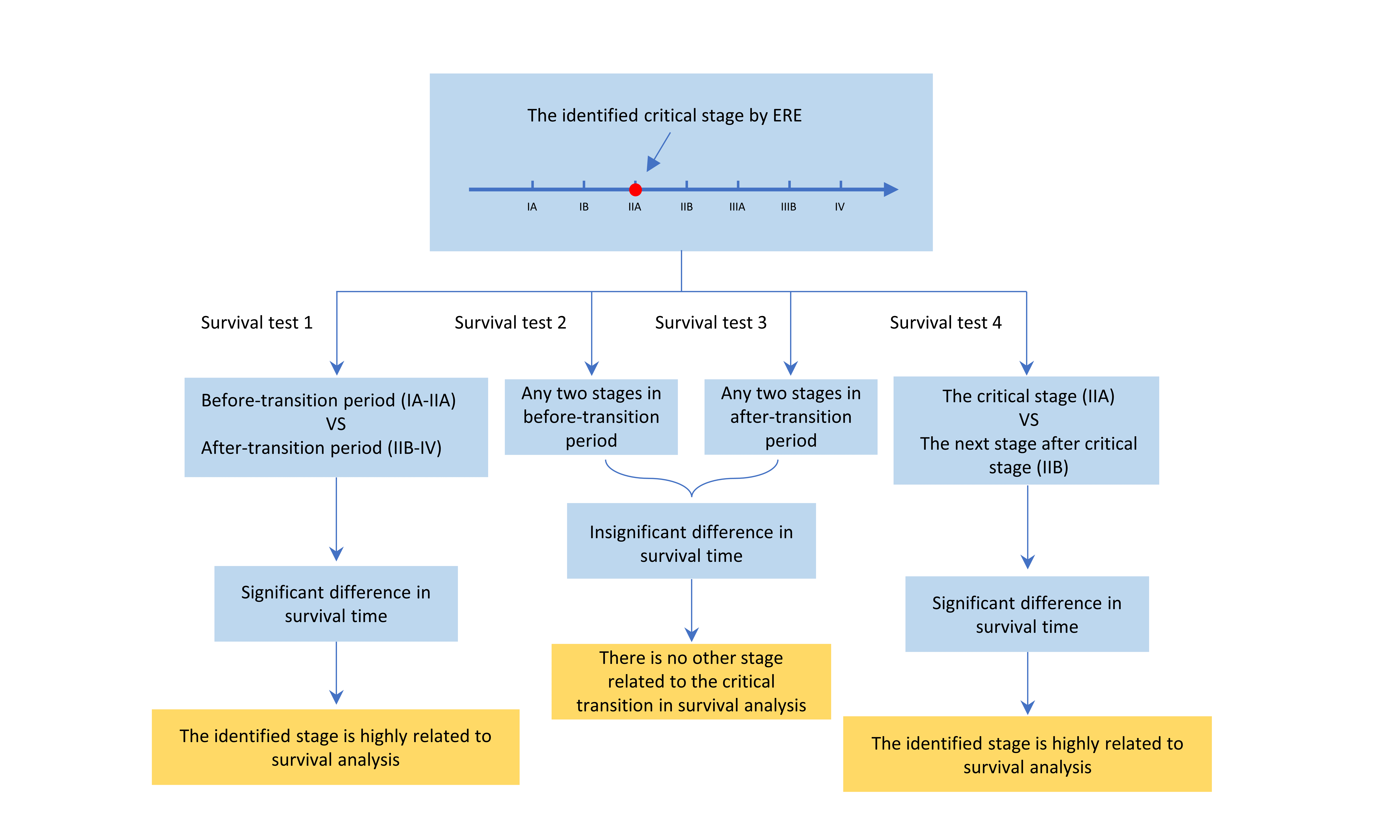
**

**Figure S3. A schematic illustration for validating the identified critical state.** To validate the identified critical state, the above four steps (survival test 1, survival test 2, survival test 3 and survival test 4) were carried out for validating a critical transition of tumor disease at stage IIA.

## Fig. S4. Validating the identified critical states of THCA and KIRP


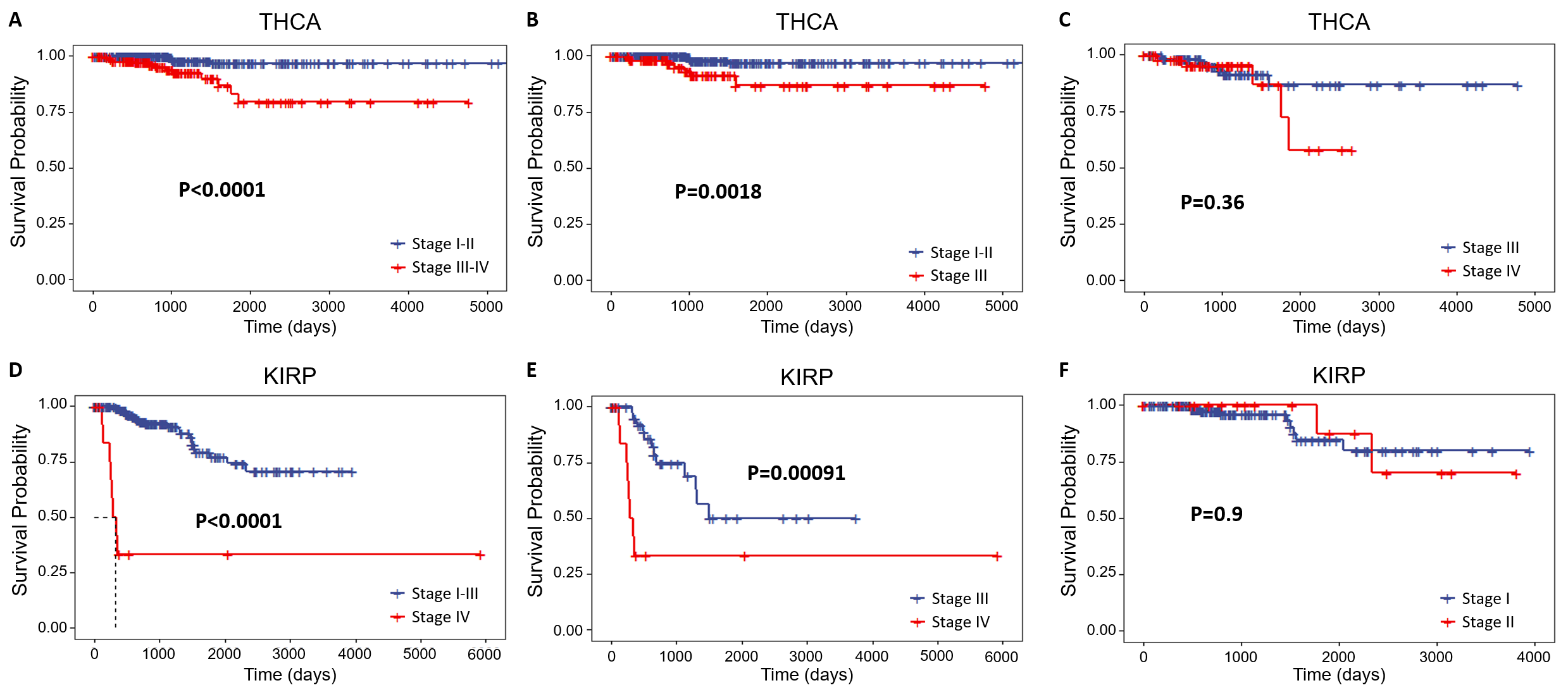


**Figure S4. Validation for the identified critical states of THCA and KIRP. (A-C)** validate the identified critical state of THCA based on the validation strategy. **(D-E)** validate the identified critical state of KIRP based on the validation strategy.

## Fig. S5. The performance of ERE under different sample sizes in numerical simulation


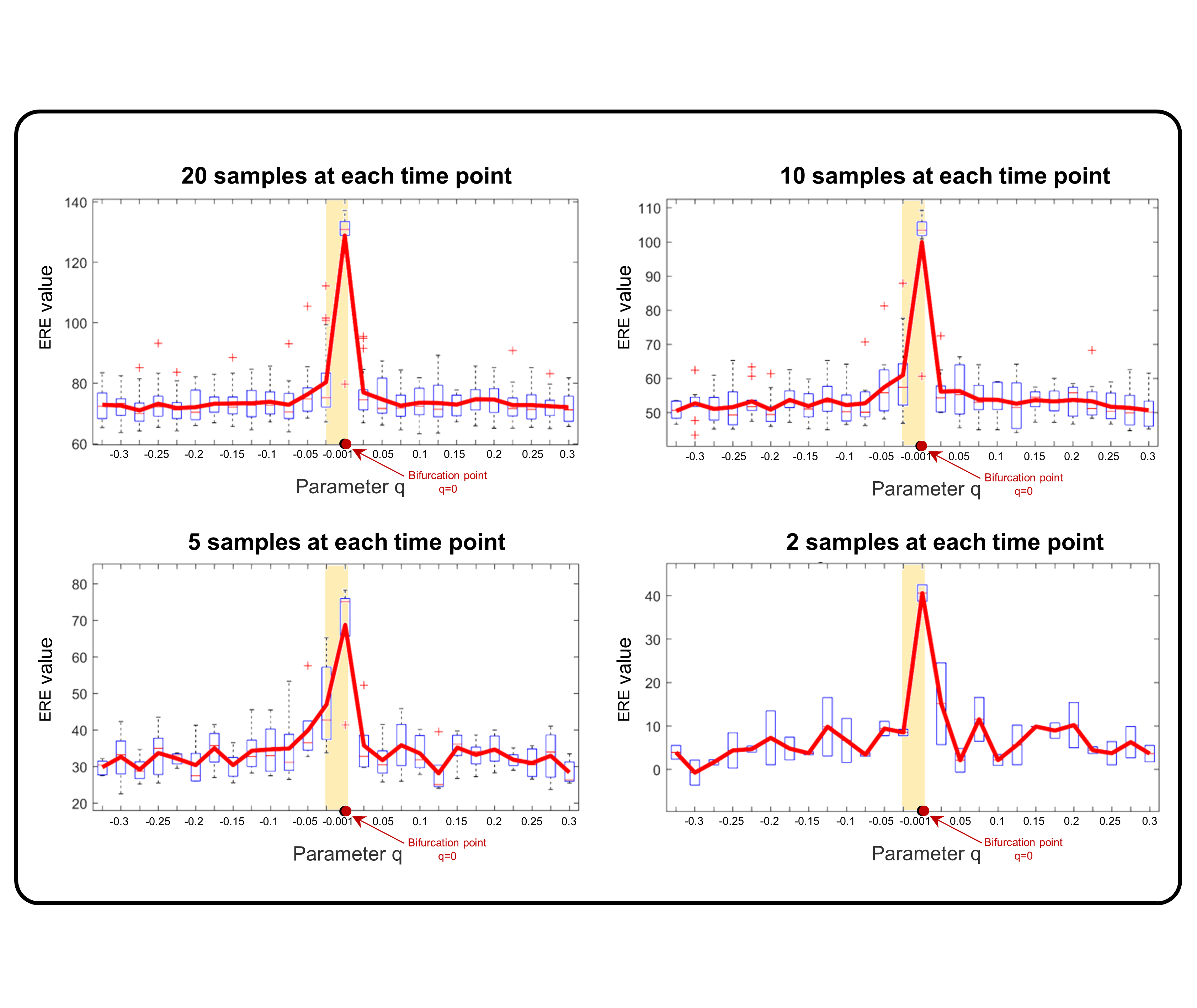


**Figure S5. The performance of ERE under different sample sizes in numerical simulation.** ERE is effective in identifying the critical points of the simulated data with different sample sizes, validating the robustness of ERE.

## Fig. S6. Comparison of the performance of ERE under different noise strengths in numerical simulation with other methods





**Figure S6.** **Comparison of the performance of ERE under different noise strengths in numerical simulation with other methods.** Even though the noise strength increases, ERE maintains a stable curve trend and provides distinct early-warning signals, validating the robustness of the ERE method.

## Fig. S7. Cancer development regulatory mechanisms revealed by ERE signaling gene pairs in KIRC


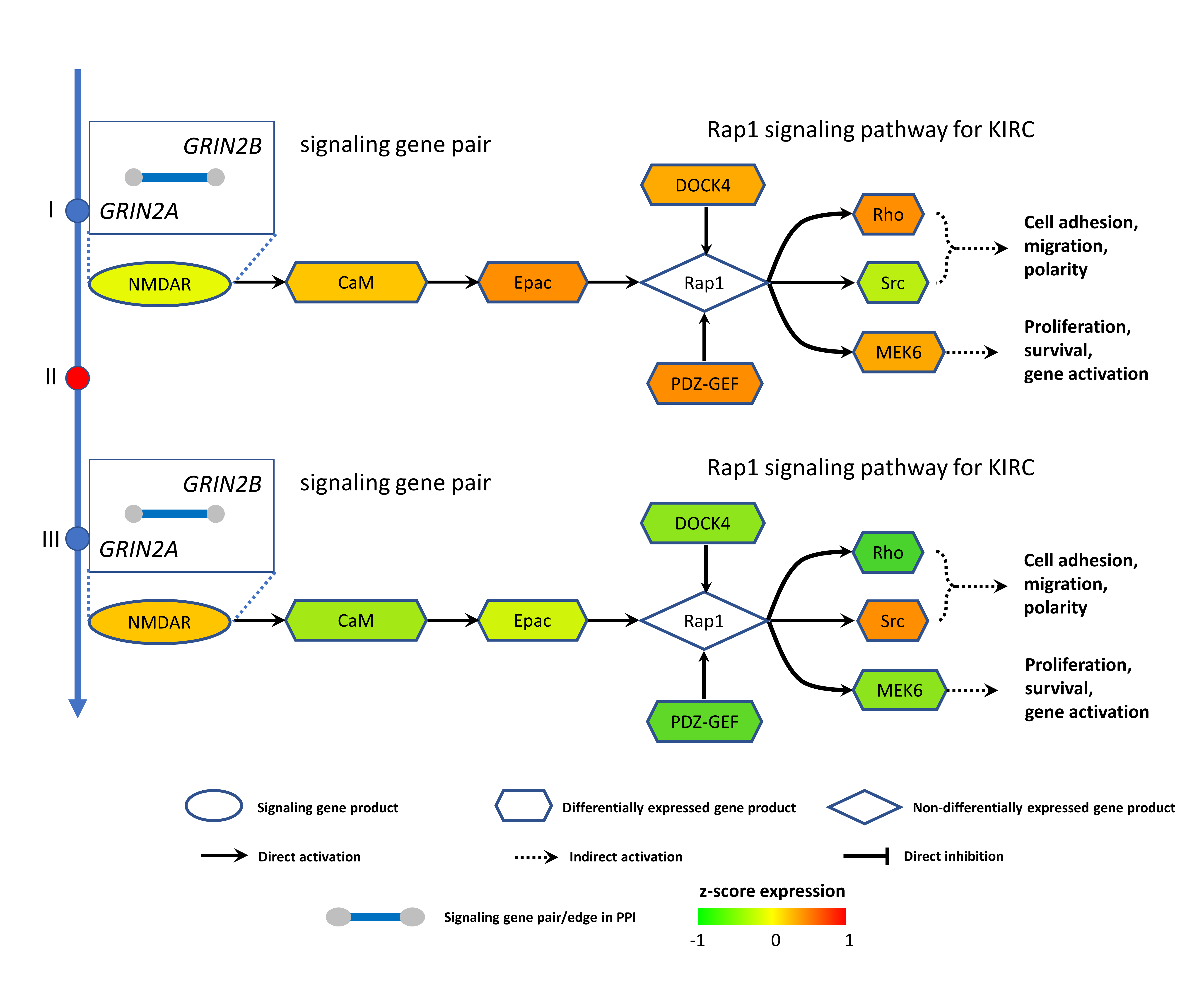


**Figure S7. Cancer development regulatory mechanisms revealed by ERE signaling gene pairs in KIRC.** Switching dynamics of downstream differential genes before and after the critical point induced by upstream ERE signaling genes in Rap1 signaling pathway for KIRC. NMDARs are coded by GRIN2A and GRIN2B, which is precisely a signaling gene pair with high ERE value.

## Fig. S8. Survival analysis based on positive and negative edge biomarkers for COAD, LUAD, THCA and KIRC





**Figure S8. Survival analysis based on positive and negative edge biomarkers for COAD, LUAD, THCA and KIRC.** **(A)** The survival expectancy of COAD patients with high ERE values in positive/ negative edge biomarkers is significantly different from those with low ERE values in the biomarkers. **(B)** The survival expectancy of LUAD patients with high ERE values in positive/ negative edge biomarkers is significantly different from those with low ERE values in the biomarkers. **(C)** The survival expectancy of THCA patients with high ERE values in positive/ negative edge biomarkers is significantly different from those with low ERE values in the biomarkers. **(D)** The survival expectancy of KIRC patients with high ERE values in positive/ negative edge biomarkers is significantly different from those with low ERE values in the biomarkers.

## Fig. S9. A model of 16-nodes network for numerical simulation


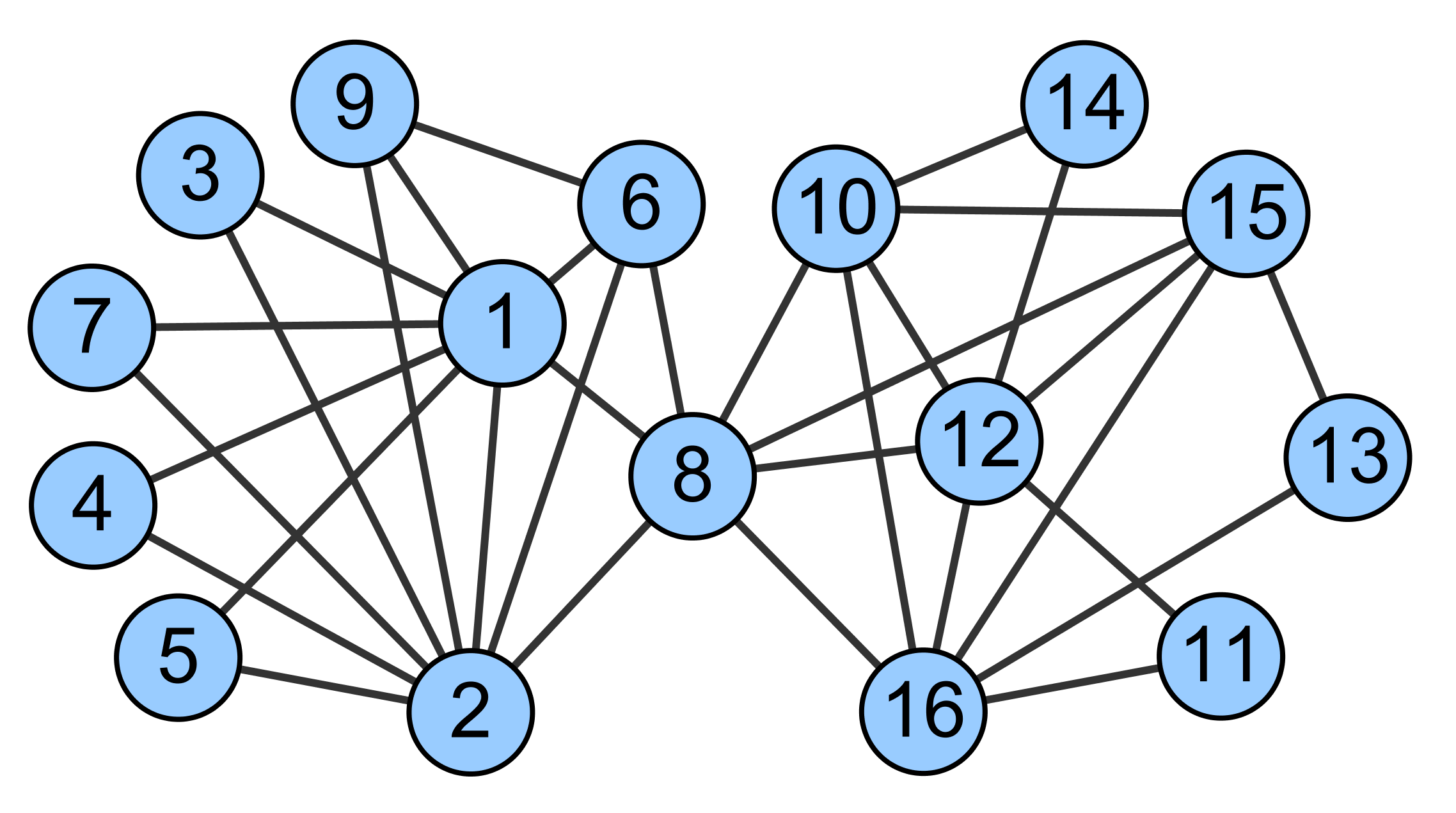


**Figure S9. A model of 16-nodes network for numerical simulation.** In the network, the nodes represent genes, and the edges represent positive or negative regulations among genes. The background differential equation set is shown as Eq. (S2).

## Fig. S10. Probability density functions fit by kernel density estimation based on normal and case samples


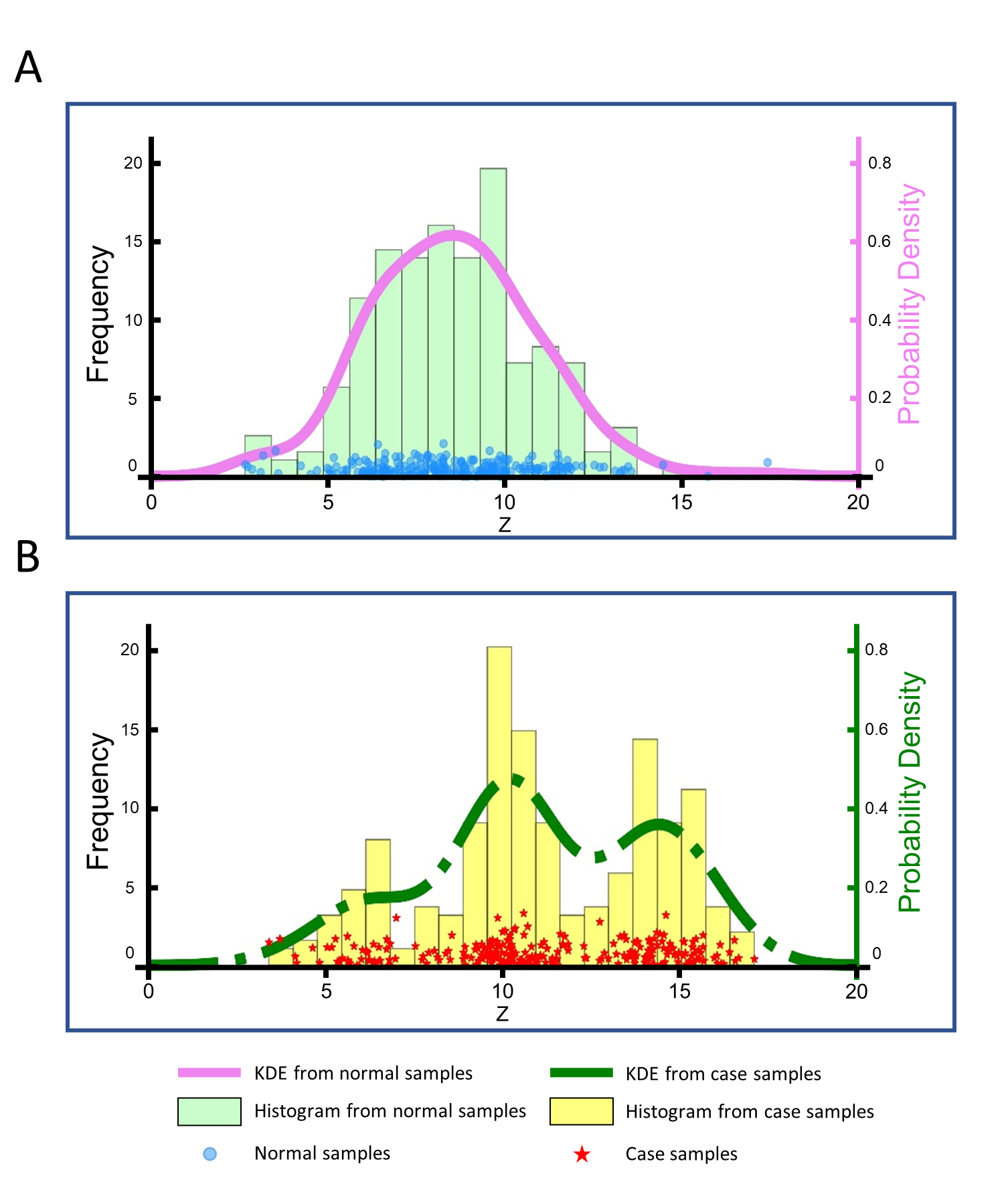


**Figure S10.** **Probability density functions fit by kernel density estimation based on normal and case samples. (A)** Probability density function fit by kernel density estimation based on the gene expression data of normal samples. **(B)** Probability density function fit by kernel density estimation based on the gene expression data of case samples. From the above figures, it can be seen that kernel density estimation is relatively effective in fitting the distribution of the data.

## Fig. S11. Comparison of the performances of the ERE method and traditional biomarkers





**Figure S11. Identifying critical stages for tumor deterioration based on the ERE method and traditional biomarkers (Table S1): (A)** COAD, **(C)** LUAD, **(E)** THCA, and **(G)** KIRC. Survival curves for patients before and after the critical stage: **(B)** COAD, (**D)** LUAD, **(F)** THCA, and **(H)** KIRC.

## Fig. S12. Dynamic evolution of the networks across all stages in each tumor


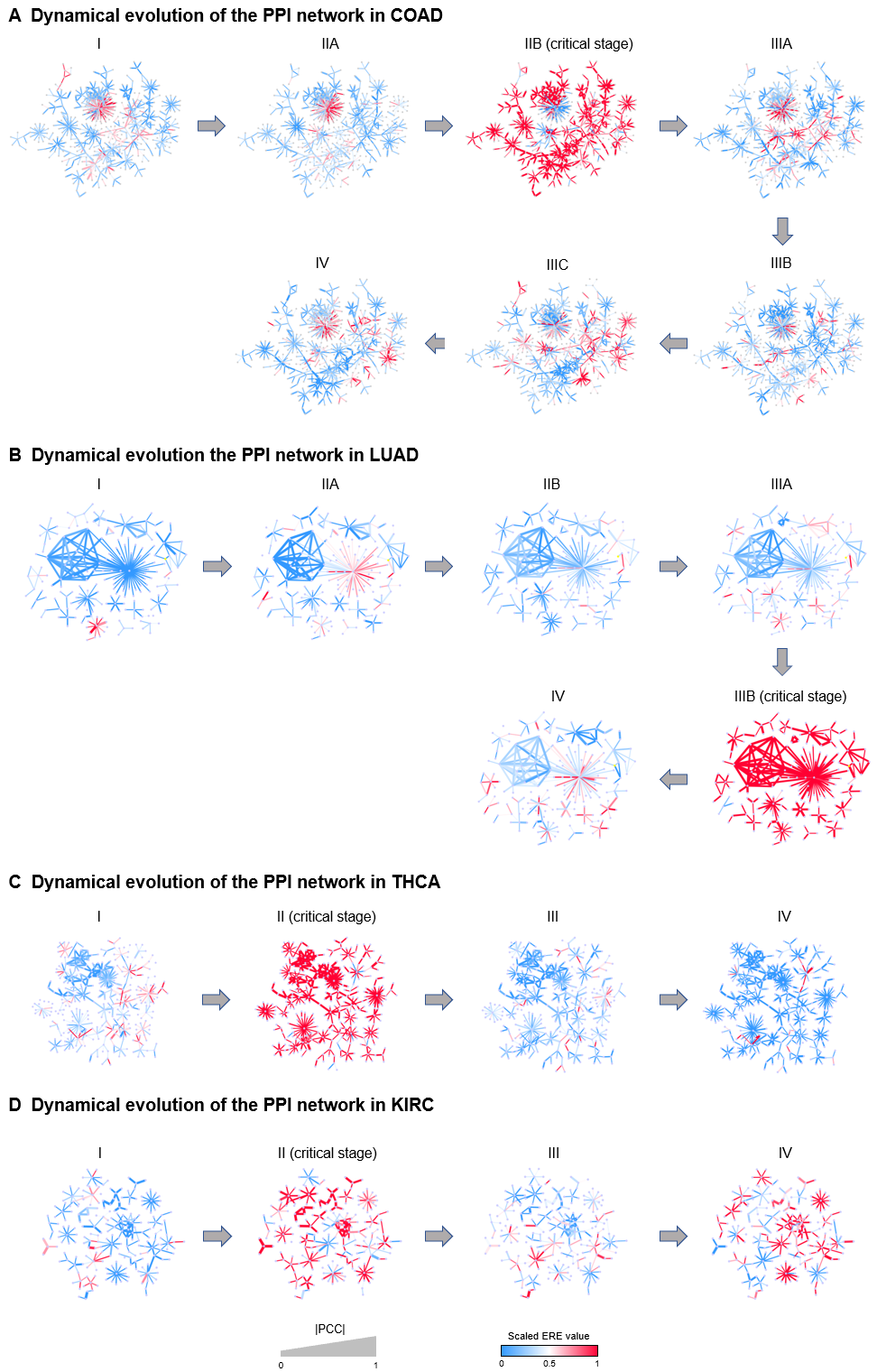


**Figure S12.** **Dynamic evolution of networks consisting of ERE signaling gene pairs across all stages in (A) COAD, (B) LUAD, (C) THCA, and (D) KIRC.**

## Fig. S13. Comparison of the prognosis results based on the identified critical stages by the ERE method and DEGs for THCA


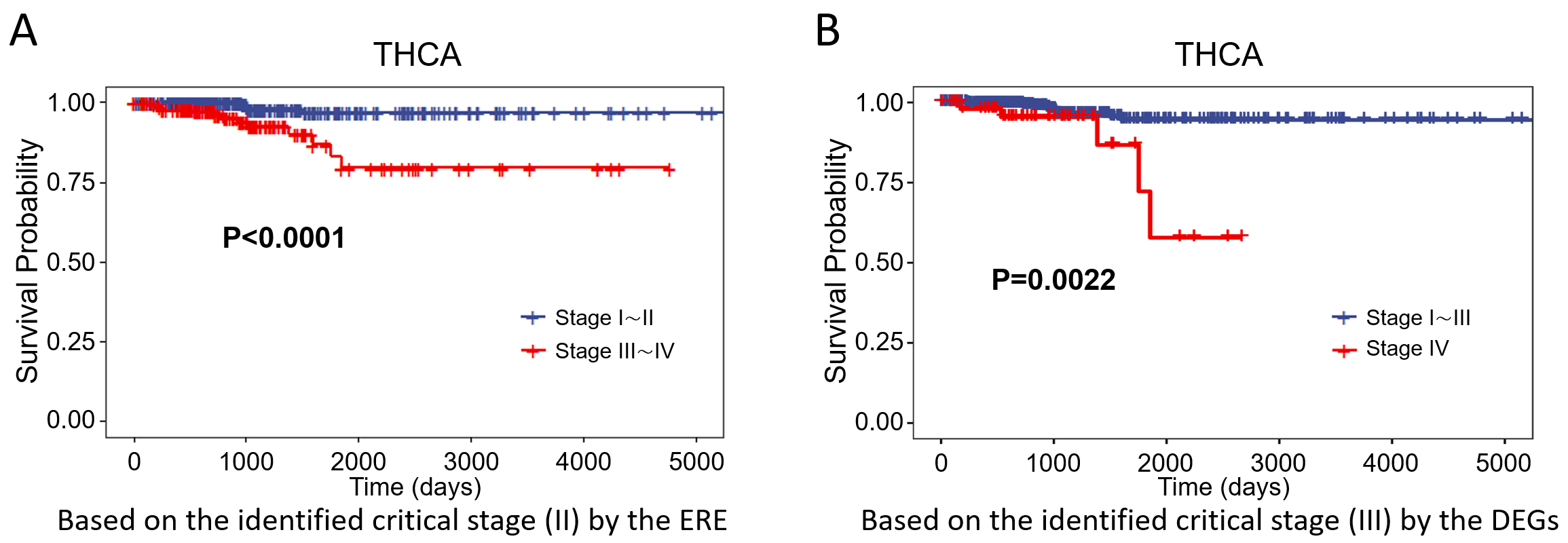


**Figure S13. Comparison of the prognosis results based on the identified critical stages by the ERE method and DEGs (differentially expressed genes) for THCA. (A)** Survival curves before and after the critical stage II (identified by the ERE method) in THCA patients ($P<0.0001$). **(B)** Survival curves before and after the critical stage III (identified by the mean expression of DEGs) in THCA patients ($P=0.0022$). Notably, there is a more significant difference between the survival curves before and after stage II than stage III, validating that there is a critical transition that leads to different survival time in stage II instead of stage III.

## Fig. S14. Performance of ERE in numerical simulation


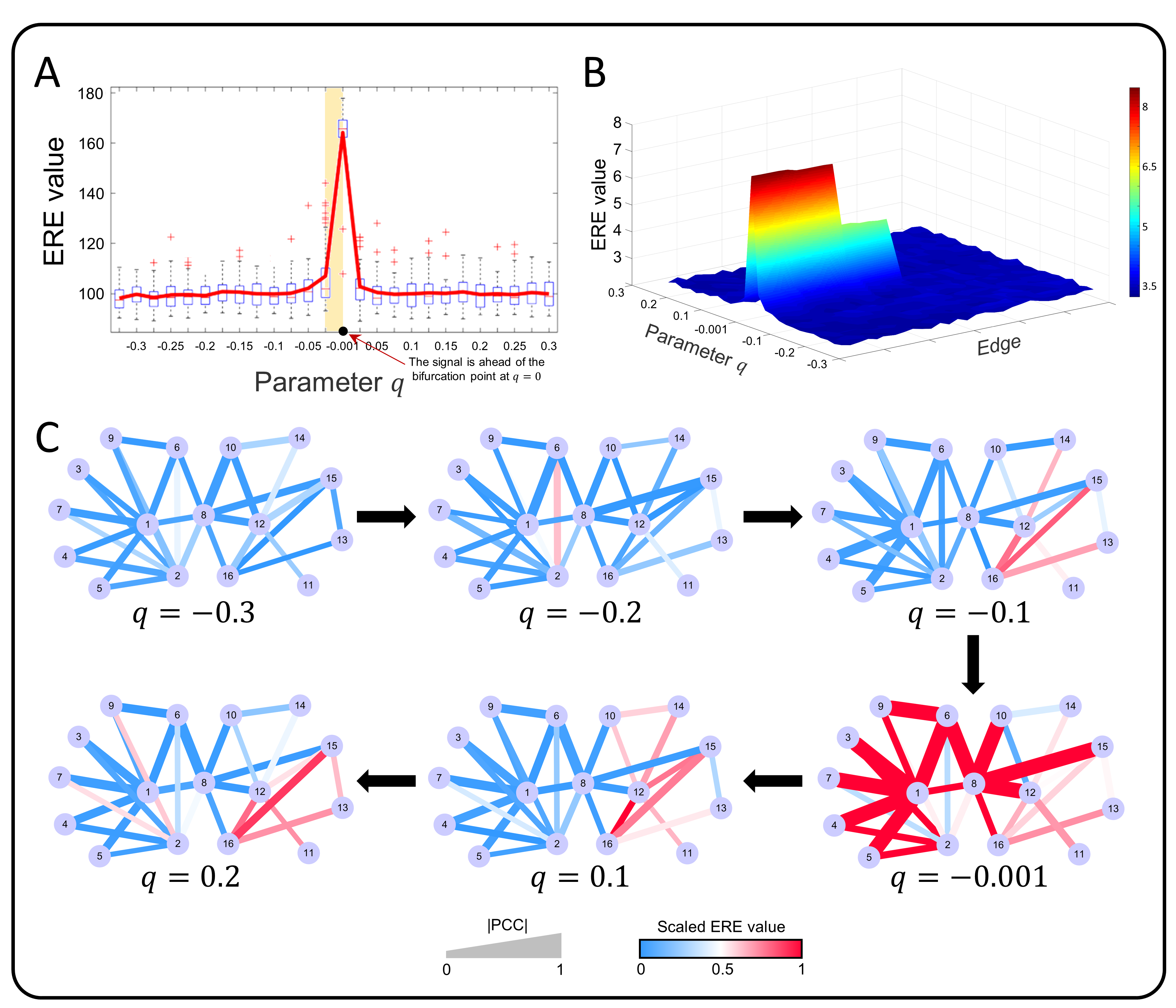


**Fig. S14. Performance of ERE illustrated by a 16-node simulated network model.** **(A)** As shown in the ERE scoring curve defined in the Method section, the ERE value increases abruptly as the system is in the vicinity of the bifurcation point $q = 0$. **(B)** The dynamic evolution of local ERE values illustrates the landscape of the edge-based relative entropy from a global perspective. **(C)** To visualize the changes in the critical period, we depict the entropy changes of gene pairs at the network level. The simulated network shows a significant change close to the bifurcation point. The color of each edge indicates the scaled ERE value, and the width indicates the absolute value of the Pearson correlation coefficient ($|PCC|$).

## Fig. S15. The performance of ERE on the COAD, LUAD, THCA, and KIRC using a bootstrapping strategy


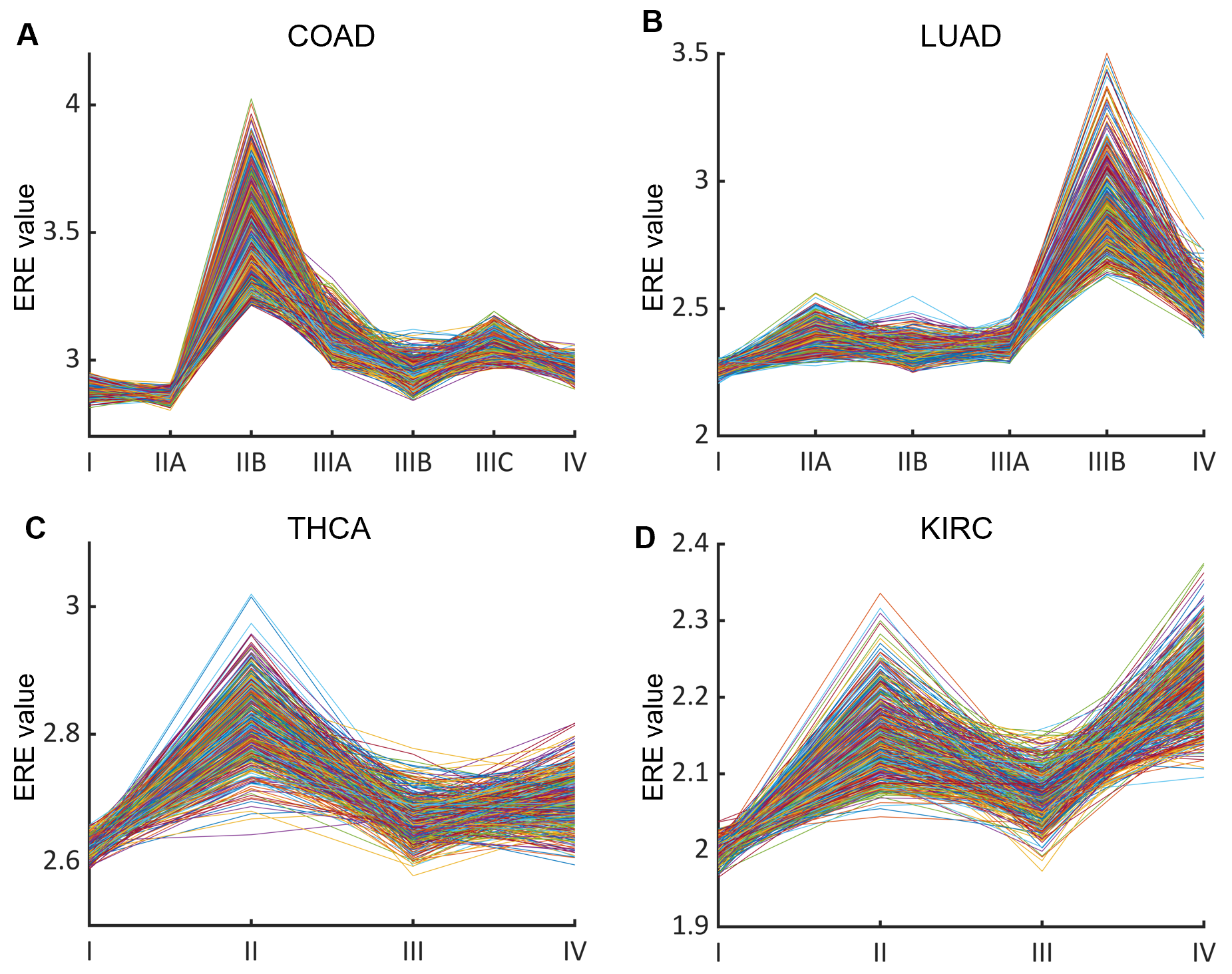


**Fig. S15. The performance of ERE on the COAD, LUAD, THCA, and KIRC using a bootstrapping strategy.** Specifically, the ERE values from each sampling iteration are summarized for each dataset, *i.e.*, (A) COAD, (B) LUAD, (C) THCA, and (D) KIRC. For each sub-figure, each curve represents the ERE values obtained from a single random sampling scheme. It is seen that the local maximum points of most (above 95%) ERE curves are consistent with the results of the manuscript.

## Fig. S16. An illustration of the “after-transition state”


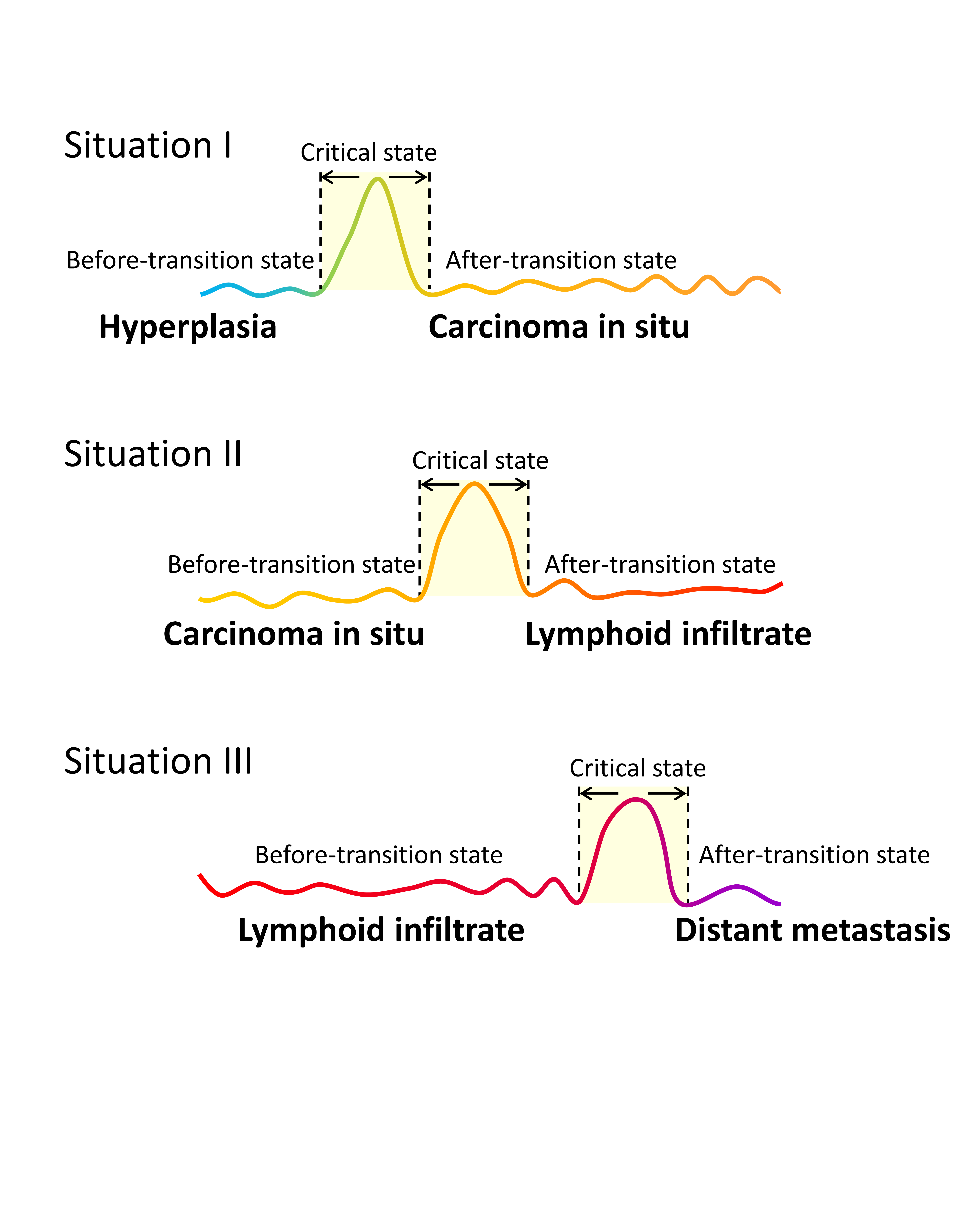


**Fig. S16. An illustration of the "after-transition state".** There may be multiple critical states occurring during disease progression. If the state after the critical state shows drastic deterioration compared to before, we consider it the “after-transition state” or the “disease state”. We take cancer as an example. (I) Patients experience a drastic deterioration at the stage of carcinoma in situ compared to the hyperplasia stage. Therefore, the stage of carcinoma in situ is considered as the “after-transition state”. (II) The lymphoid infiltrates are more severe deterioration compared to the stage of carcinoma in situ, and is considered as the “after-transition state”. (III) The stage of distant metastasis is a more severe stage compared to the stage of lymphoid infiltrates, almost incurable, and is considered as the “after-transition state”.

## Fig. S17. The three stages transition of interactions between certain genes during the entire disease progression


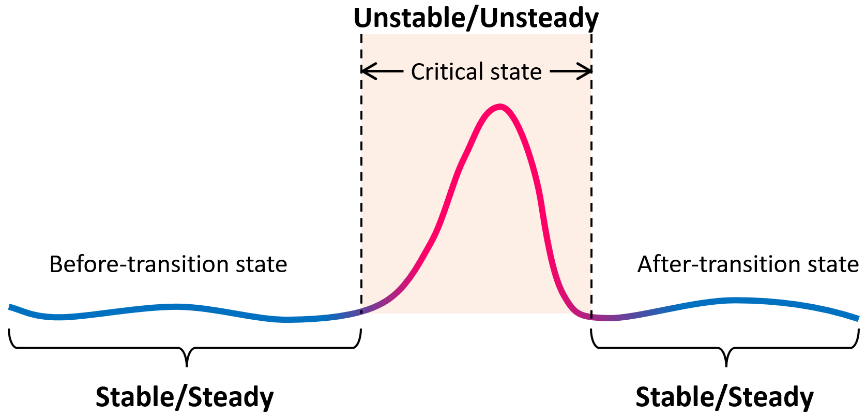


**Fig. S17. The three stages transition of interactions between certain genes during the entire disease progression.** Specifically, some interactions between certain genes (nodes) may exhibit instability in the critical state, while they remain stable both in the before-transition and after-transition states.

## Fig. S18. The comparison of the performance of ERE with the standard relative entropy


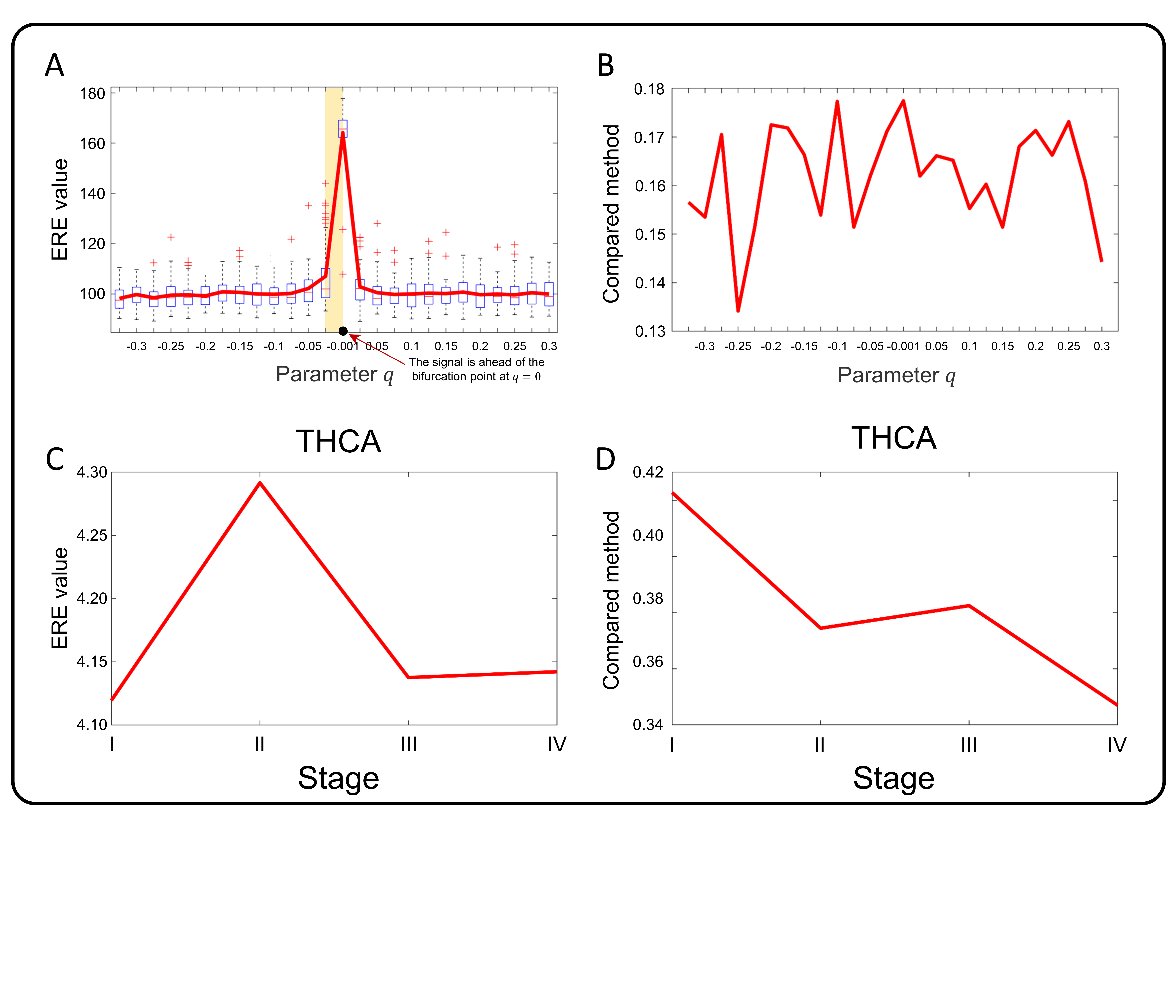


**Fig. S18. The comparison of the performance of ERE in the simulated dataset and THCA with the standard relative entropy.** ERE effectively signals the critical points of the simulated data and THCA in (A) and (C), respectively. However, it fails to capture the early-warning signals in (B) and (D) under the standard relative entropy.

## Fig. S19. Box plot of ERE values for acute lung injury


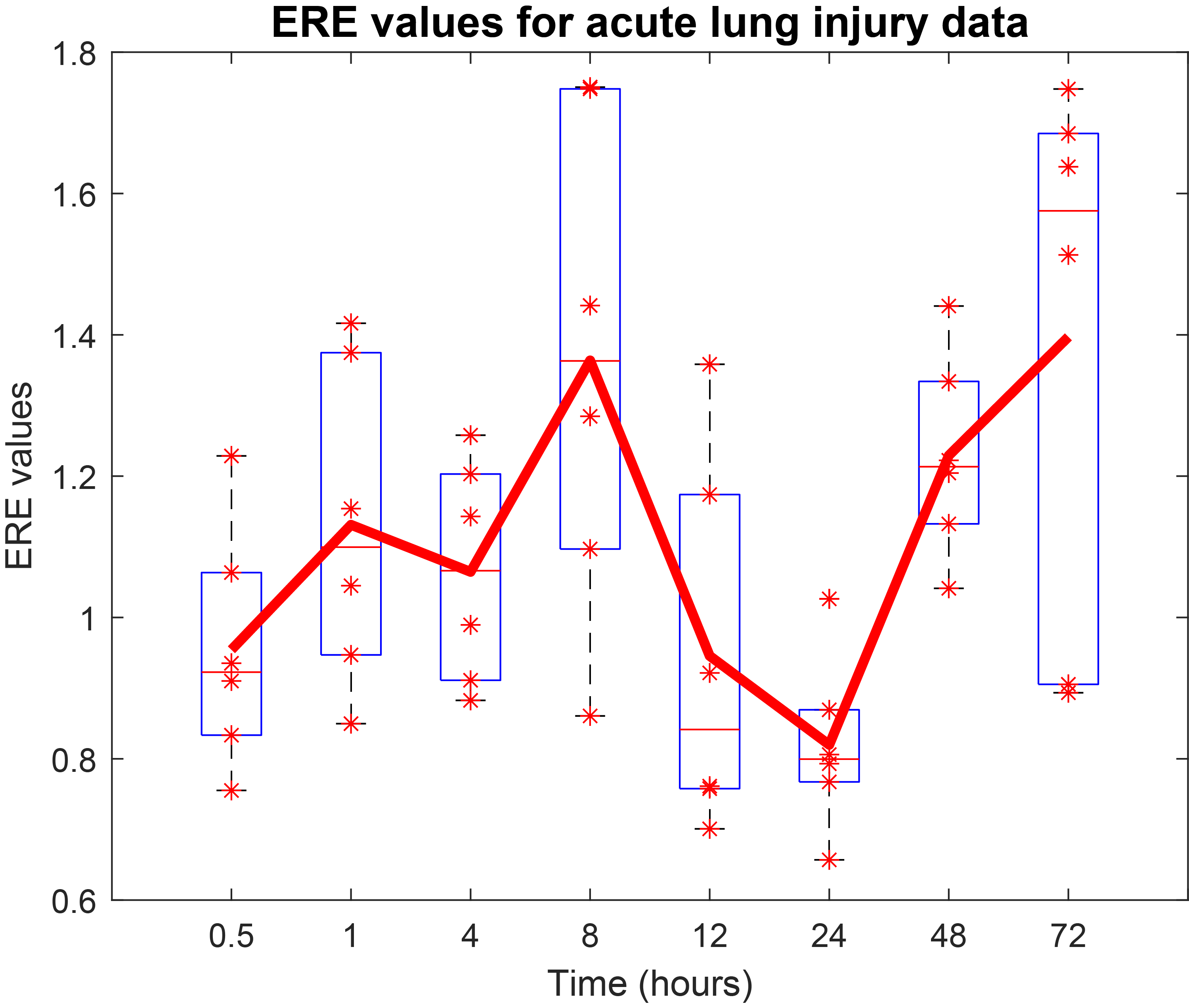


**Fig. S19. Box plot illustrating the variability of the ERE value at each time point in acute lung injury.** The sample-specific ERE values exhibit variations within a certain range at each time point due to the intrinsic specificity of individual samples.

## Fig. S20. The performance of ERE under different groups of edges with highest ERE values





**Fig. S20. The performance of ERE under different groups of edges with highest ERE values on simulated data and THCA data.** We compared the performance of ERE based on the modification on the step 3 of the "Materials and Methods " section, that is, different groups of edges with highest ERE values (top 5%, 10%, 20%, and 50%). The consistent trends of the resulting ERE curves and successful detection of critical points in both simulated and THCA datasets demonstrated the stability and robustness of ERE across various high-ERE value edge sets.

## Fig. S21. Comparison of the performance of ERE with that of pure physical approaches


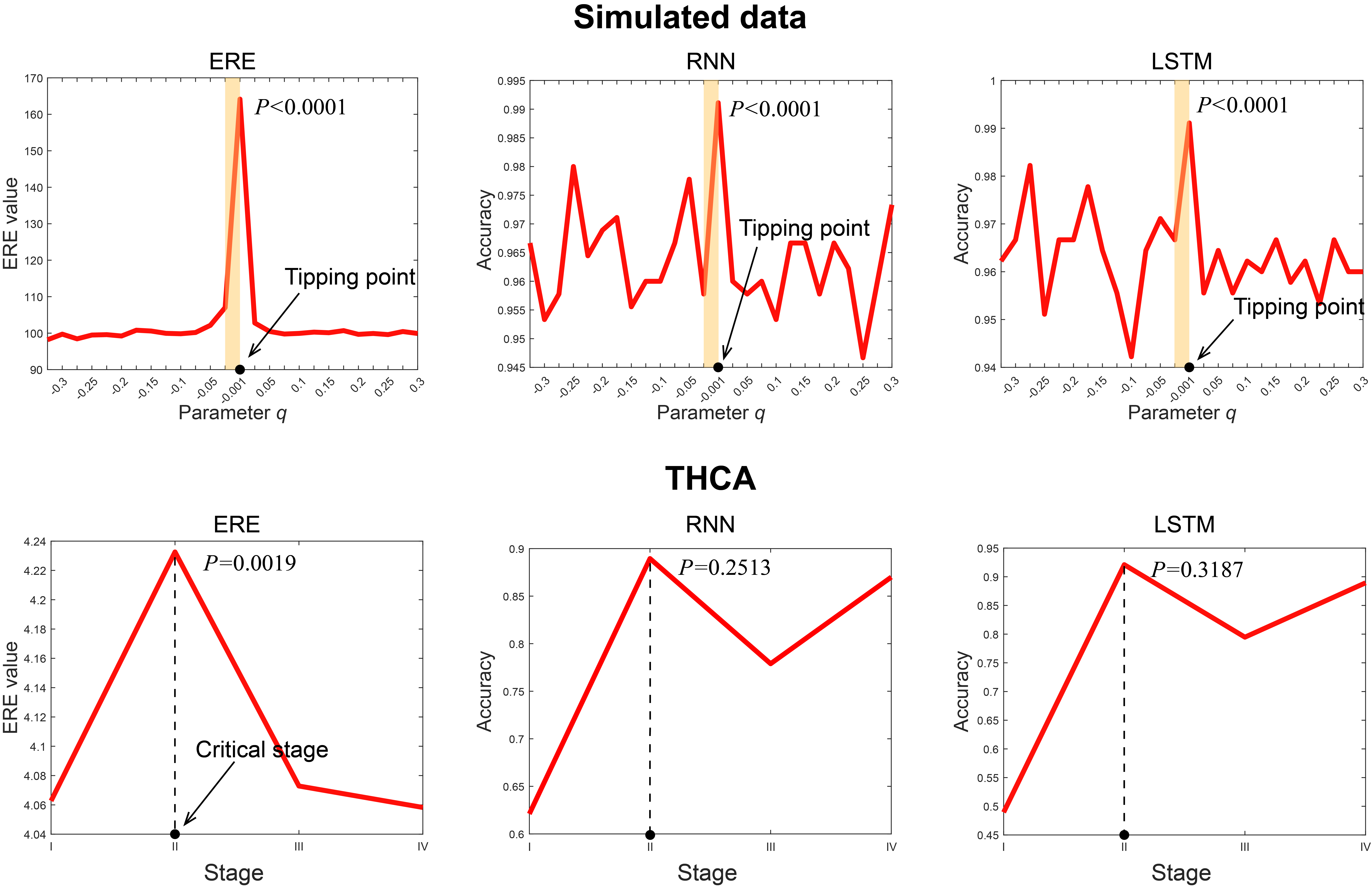


**Fig. S21. Comparison of the critical point detection performance of ERE in simulated and THCA datasets with that of two pure physical approaches (RNN and LSTM).** The performance of ERE, RNN, and LSTM on simulated data and THCA is summarized in the left, middle, and right panels, respectively.

**Section S3. The Supplementary Notes**

## Supplementary Note S1. Details of kernel density estimation

The kernel density estimation is a mathematic process of finding an estimate probability density function of a random variable [5-8]. The estimation attempts to infer characteristics of a population, based on a finite data set. The data smoothing problem often is used in signal processing and data science, as it is a powerful way to estimate probability density. In short, the technique allows one to create a smooth curve given a set of random data.

Let $\{z_{1},z_{2},...,z_{N}\}$ be independent and identically distributed observations taken from some univariate distribution with an unknown probability density function$f$. For any given point$z$, its kernel density estimator is:

$f_{h}(z)=\frac{1}{N}\sum_{i=1}^{N} K_{h}(z-z_{i})=\frac{1}{Nh}\sum_{i=1}^{N} K(\frac{z-z_{i}}{h})$, (S1)

where $K$ is the kernel, a nonnegative function, and $h > 0$ is a smoothing parameter called the bandwidth. In this study, the Gaussian density function is used as the kernel, and the default bandwidth is most suitable for normal distribution density, *i.e.*, $h=\left( \frac{4\sigma^{5}}{3N} \right)^{\frac{1}{5}}$ [8, 9], where $\sigma$ is the standard deviation of samples. The probability density functions fit by kernel density estimation based on normal and case samples are characterized in Fig. S10.

The imbalance in the number of reference group samples and case group samples also does not affect the calculation of the ERE method. Our algorithm aims to detect dynamic changes at the individual level in gene interactions represented by network edges. More specifically, it is designed to identify dynamic differences between the expression of a gene $i$ in each individual within the case group and the overall expression distribution of its partner gene $j$ in the reference group. If we adhere to the standard definition of relative entropy (that is, evaluate $p_{r}$ and $p_{c}$ at the same value within the integral), we can only obtain a scalar representing the overall expression distribution difference at a time point, failing to capture the individual-level changes in gene interactions. Therefore, we modified the standard representation of relative entropy by evaluating $p_{r}$ and $p_{c}$ at different points to quantify the individual-level dynamic changes. Furthermore, we conducted a comparison of our method on both the simulated dataset and a real dataset (*i.e.*, THCA) with the standard relative entropy. The result (Fig. S18) indicates the effectiveness and robustness of our method in signaling critical points.

## Supplementary Note S2. Details of numerical simulation

We use a sixteen-gene network to conduct a numerical simulation and theoretically demonstrate the detection of early-warning signals through the ERE scheme [10]. The following sixteen differential equations represent the gene regulation of sixteen genes in a network where gene regulation is represented in a Michaelis-Menten form with the change rates, which are often used to study various biological processes including transcription, translation, diffusion, and translocation processes [11-15]. The differential equation set is as follows:

$\left\{ \begin{aligned} &\frac{dz_{1}(t)}{dt}=\frac{(8-4q)z_{2}(t)}{15(1+z_{2}(t))}-\frac{4\left( 1+q \right)}{15}z_{1}\left( t \right)+\zeta_{1}(t) \\ &\frac{dz_{2}(t)}{dt}=\frac{(4-2q)z_{1}(t)}{15(1+z_{1}(t))}-\frac{2\left( 1+q \right)}{15}z_{2}\left( t \right)+\zeta_{2}(t) \\ &\frac{dz_{3}(t)}{dt}=\frac{4q-10}{15}+\frac{5-2q}{15(1+z_{1}(t))}+\frac{5-2q}{15(1+z_{2}(t))}-z_{3}\left( t \right)+\zeta_{3}(t) \\ &\frac{dz_{4}(t)}{dt}=\frac{(6-2q)z_{1}(t)}{15(1+z_{1}(t))}+\frac{(6-2q)z_{2}(t)}{15(1+z_{2}(t))}-\frac{6}{5}z_{4}\left( t \right)+\zeta_{4}(t) \\ &\frac{dz_{5}(t)}{dt}=\frac{4q-14}{15}+\frac{7-2q}{15(1+z_{1}(t))}+\frac{7-2q}{15(1+z_{2}(t))}-\frac{7}{5}z_{5}\left( t \right)+\zeta_{5}(t) \\ &\frac{dz_{6}(t)}{dt}=\frac{4q-16}{15}+\frac{2(4-2q)}{15(1+z_{1}(t))}+\frac{2(4-2q)}{15(1+z_{2}(t))}-\frac{8}{5}z_{6}\left( t \right)+\zeta_{6}(t) \\ &\frac{dz_{7}(t)}{dt}=\frac{(9-2q)z_{1}(t)}{15(1+z_{1}(t))}+\frac{(9-2q)z_{2}(t)}{15(1+z_{2}(t))}-\frac{9}{5}z_{7}\left( t \right)+\zeta_{7}(t) \\ &\frac{dz_{8}\left( t \right)}{dt}=-\frac{13}{15}+\frac{2}{15\left( 1+z_{1}\left( t \right) \right)}+\frac{2}{15\left( 1+z_{2}\left( t \right) \right)}+\frac{2}{5\left( 1+z_{6}\left( t \right) \right)}+\frac{2z_{10}\left( t \right)}{5\left( 1+z_{10}\left( t \right) \right)} \\ +\frac{3z_{12}\left( t \right)}{5\left( 1+z_{12}\left( t \right) \right)}+\frac{z_{15}\left( t \right)}{5\left( 1+z_{15}\left( t \right) \right)}+\frac{1}{5\left( 1+z_{16}\left( t \right) \right)}-2z_{8}\left( t \right)+\zeta_{8}\left( t \right) \\ &\frac{dz_{9}\left( t \right)}{dt}=-1+\frac{1}{5\left( 1+z_{1}\left( t \right) \right)}+\frac{1}{5\left( 1+z_{2}\left( t \right) \right)}+\frac{3}{5\left( 1+z_{6}\left( t \right) \right)}-\frac{11}{5}z_{9}\left( t \right)+\zeta_{9}(t) \\ &\frac{dz_{10}\left( t \right)}{dt}=\frac{3z_{12}\left( t \right)}{5\left( 1+z_{12}\left( t \right) \right)}-\frac{12}{5}z_{10}\left( t \right)+\zeta_{10}(t) \\ &\frac{dz_{11}\left( t \right)}{dt}=\frac{z_{12}\left( t \right)}{4\left( 1+z_{12}\left( t \right) \right)}-\frac{13}{5}z_{11}\left( t \right)+\zeta_{11}(t) \\ &\frac{dz_{12}\left( t \right)}{dt}=-\frac{2}{5}+\frac{2z_{15}\left( t \right)}{5\left( 1+z_{15}\left( t \right) \right)}+\frac{2}{5\left( 1+z_{16}\left( t \right) \right)}-\frac{14}{5}z_{12}\left( t \right)+\zeta_{12}(t) \\ &\frac{dz_{13}\left( t \right)}{dt}=-\frac{24}{5}+\frac{1}{1+z_{15}\left( t \right)}+\frac{19}{5\left( 1+z_{16}\left( t \right) \right)}-5z_{13}\left( t \right)+\zeta_{13}(t) \\ &\frac{dz_{14}\left( t \right)}{dt}=-\frac{8}{5}+\frac{4}{5\left( 1+z_{10}\left( t \right) \right)}+\frac{4}{5\left( 1+z_{12}\left( t \right) \right)}-\frac{16}{5}z_{14}\left( t \right)+\zeta_{14}(t) \\ &\frac{dz_{15}\left( t \right)}{dt}=\frac{z_{16}\left( t \right)}{10\left( 1+z_{16}\left( t \right) \right)}-\frac{7}{2}z_{15}\left( t \right)+\zeta_{15}(t) \\ &\frac{dz_{16}\left( t \right)}{dt}=\frac{z_{15}\left( t \right)}{10\left( 1+z_{15}\left( t \right) \right)}-\frac{7}{2}z_{16}\left( t \right)+\zeta_{16}(t) \end{aligned} \right.$ (S2)

where *q* is a scalar control parameter and $\zeta_{i}(t)$ $(i = 1, 2, \ldots, 16$) are Gaussian noises with zero means. $z_{i}(t)$($i = 1, 2, \ldots, 16$) represent the data of monitoring station $i$. In Eq. (S2), the change rates of degradation rates of mRNAs are ($-4\frac{1+q}{15}$, $-2\frac{4+q}{15}$, $-1$, $-\frac{6}{5}$, $-\frac{7}{5}$, $-\frac{8}{5}$, $-\frac{9}{5}$, $-2$, $-\frac{11}{5}$, $-\frac{12}{5}$, $-\frac{13}{5}$, $-\frac{14}{5}$, $-3$, $-\frac{16}{5}$, $-\frac{7}{2}$, $-\frac{7}{2})$. The stable equilibrium point $\bar{Z}=(\bar{z}_{1}, \bar{z}_{2}, \ldots, \bar{z}_{16}) =(0, 0, \ldots, 0)$. The differential equations Eq. (S2) can be transformed into the difference equations $Z\left( k+1 \right)=f(Z\left( k \right),q)$ with a small time interval $\Delta t$ using the Euler scheme [16], *i.e.*,

$\left\{ \begin{aligned} &z_{1}(k+1)=z_{1}\left( k \right)+\left[ \frac{\left( 8-4q \right)z_{2}\left( k \right)}{15\left( 1+z_{2}\left( k \right) \right)}-\frac{4\left( 1+q \right)}{15}z_{1}\left( k \right)+\zeta_{1}\left( k \right) \right]\Delta t \\ &z_{2}(k+1)=z_{2}\left( k \right)+\left[ \frac{(4-2q)z_{1}(k)}{15(1+z_{1}(k))}-\frac{2\left( 1+q \right)}{15}z_{2}\left( k \right)+\zeta_{2}(k) \right]\Delta t \\ &z_{3}(k+1)=z_{3}\left( k \right)+\left[ \frac{4q-10}{15}+\frac{5-2q}{15\left( 1+z_{1}\left( k \right) \right)}+\frac{5-2q}{15\left( 1+z_{2}\left( k \right) \right)}-z_{3}\left( k \right)+\zeta_{3}\left( k \right) \right]\Delta t \\ &z_{4}(k+1)=z_{4}\left( k \right)+\left[ \frac{(6-2q)z_{1}(k)}{15(1+z_{1}(k))}+\frac{(6-2q)z_{2}(k)}{15(1+z_{2}(k))}-\frac{6}{5}z_{4}\left( k \right)+\zeta_{4}(k) \right]\Delta t \\ &z_{5}(k+1)=z_{5}\left( k \right)+\left[ \frac{4q-14}{15}+\frac{7-2q}{15(1+z_{1}(k))}+\frac{7-2q}{15(1+z_{2}(k))}-\frac{7}{5}z_{5}\left( k \right)+\zeta_{5}(k) \right]\Delta t \\ &z_{6}(k+1)=z_{6}\left( k \right)+\left[ \frac{4q-16}{15}+\frac{2(4-2q)}{15(1+z_{1}(k))}+\frac{2(4-2q)}{15(1+z_{2}(k))}-\frac{8}{5}z_{6}\left( k \right)+\zeta_{6}(k) \right]\Delta t \\ &z_{7}(k+1)=z_{7}\left( k \right)+\left[ \frac{(9-2q)z_{1}(k)}{15(1+z_{1}(k))}+\frac{(9-2q)z_{2}(k)}{15(1+z_{2}(k))}-\frac{9}{5}z_{7}\left( k \right)+\zeta_{7}(k) \right]\Delta t \\ &z_{8}(k+1)=z_{8}\left( k \right)+\left[ -\frac{13}{15}+\frac{2}{15\left( 1+z_{1}\left( k \right) \right)}+\frac{2}{15\left( 1+z_{2}\left( k \right) \right)}+\frac{2}{5\left( 1+z_{6}\left( k \right) \right)}+\frac{2z_{10}\left( k \right)}{5\left( 1+z_{10}\left( k \right) \right)} \right. \\ \left. +\frac{3z_{12}(k)}{5(1+z_{12}(k))}+\frac{z_{15}\left( k \right)}{5\left( 1+z_{15}\left( k \right) \right)}+\frac{1}{5\left( 1+z_{16}\left( k \right) \right)}-2z_{8}\left( k \right)+\zeta_{8}(k) \right]\Delta t \\ &z_{9}(k+1)=z_{9}\left( k \right)+\left[ -1+\frac{1}{5\left( 1+z_{1}\left( k \right) \right)}+\frac{1}{5\left( 1+z_{2}\left( k \right) \right)}+\frac{3}{5\left( 1+z_{6}\left( k \right) \right)}-\frac{11}{5}z_{9}\left( k \right)+\zeta_{9}(k) \right]\Delta t \\ &z_{10}(k+1)=z_{10}\left( k \right)+\left[ \frac{3z_{12}\left( k \right)}{5\left( 1+z_{12}\left( k \right) \right)}-\frac{12}{5}z_{10}\left( k \right)+\zeta_{10}(k) \right]\Delta t \\ &z_{11}(k+1)=z_{11}\left( k \right)+\left[ \frac{z_{12}\left( k \right)}{4\left( 1+z_{12}\left( k \right) \right)}-\frac{13}{5}z_{11}\left( k \right)+\zeta_{11}(k) \right]\Delta t \\ &z_{12}(k+1)=z_{12}\left( k \right)+\left[ -\frac{2}{5}+\frac{2z_{15}\left( k \right)}{5\left( 1+z_{15}\left( k \right) \right)}+\frac{2}{5\left( 1+z_{16}\left( k \right) \right)}-\frac{14}{5}z_{12}\left( k \right)+\zeta_{12}(k) \right]\Delta t \\ &z_{13}(k+1)=z_{13}\left( k \right)+\left[ -\frac{24}{5}+\frac{1}{1+z_{15}\left( k \right)}+\frac{19}{5\left( 1+z_{16}\left( k \right) \right)}-5z_{13}\left( k \right)+\zeta_{13}(k) \right]\Delta t \\ &z_{14}(k+1)=z_{14}\left( k \right)+\left[ -\frac{8}{5}+\frac{4}{5\left( 1+z_{10}\left( k \right) \right)}+\frac{4}{5\left( 1+z_{12}\left( k \right) \right)}-\frac{16}{5}z_{14}\left( k \right)+\zeta_{14}(k) \right]\Delta t \\ &z_{15}(k+1)=z_{15}\left( k \right)+\left[ \frac{z_{16}\left( k \right)}{10\left( 1+z_{16}\left( k \right) \right)}-\frac{7}{2}z_{15}\left( k \right)+\zeta_{15}(k) \right]\Delta t \\ &z_{16}(k+1)=z_{16}\left( k \right)+\left[ \frac{z_{15}\left( k \right)}{10\left( 1+z_{15}\left( k \right) \right)}-\frac{7}{2}z_{16}\left( k \right)+\zeta_{16}(k) \right]\Delta t \end{aligned} \right.$ (S3)

Note that $Z(k)$ is the vector of $Z(t)$ at the time instant $t=k\Delta t$.The Jacobian matrix of Eq. (S2) is denoted as $J=\frac{\partial f(Z;q)}{\partial Z}│_{Z=\bar{Z}}$, where

$J=e^{\Delta t\cdot A}$ (S4)

with

$A=\left[ \begin{matrix} \frac{-4-4q}{15} & \frac{8-4q}{15} & 0 & 0 & 0 & 0 & 0 & 0 & 0 & 0 & 0 & 0 & 0 & 0 & 0 & 0 \\ \frac{4-2q}{15} & \frac{-8-2q}{15} & 0 & 0 & 0 & 0 & 0 & 0 & 0 & 0 & 0 & 0 & 0 & 0 & 0 & 0 \\ \frac{-5+2q}{15} & \frac{-5+2q}{15} & -1 & 0 & 0 & 0 & 0 & 0 & 0 & 0 & 0 & 0 & 0 & 0 & 0 & 0 \\ \frac{6-2q}{15} & \frac{6-2q}{15} & 0 & -\frac{6}{5} & 0 & 0 & 0 & 0 & 0 & 0 & 0 & 0 & 0 & 0 & 0 & 0 \\ \frac{-7+2q}{15} & \frac{-7+2q}{15} & 0 & 0 & -\frac{7}{5} & 0 & 0 & 0 & 0 & 0 & 0 & 0 & 0 & 0 & 0 & 0 \\ \frac{-8+2q}{15} & \frac{-8+2q}{15} & 0 & 0 & 0 & -\frac{8}{5} & 0 & 0 & 0 & 0 & 0 & 0 & 0 & 0 & 0 & 0 \\ \frac{9-2q}{15} & \frac{9-2q}{15} & 0 & 0 & 0 & 0 & -\frac{9}{5} & 0 & 0 & 0 & 0 & 0 & 0 & 0 & 0 & 0 \\ -\frac{2}{15} & -\frac{2}{15} & 0 & 0 & 0 & -\frac{2}{5} & 0 & -2 & 0 & \frac{2}{5} & 0 & \frac{3}{5} & 0 & \frac{1}{5} & 0 & -\frac{1}{5} \\ -\frac{1}{5} & -\frac{1}{5} & 0 & 0 & 0 & -\frac{3}{5} & 0 & 0 & -\frac{11}{5} & 0 & 0 & 0 & 0 & 0 & 0 & 0 \\ 0 & 0 & 0 & 0 & 0 & 0 & 0 & 0 & 0 & -\frac{12}{5} & 0 & \frac{3}{5} & 0 & 0 & 0 & 0 \\ 0 & 0 & 0 & 0 & 0 & 0 & 0 & 0 & 0 & 0 & -\frac{13}{5} & \frac{1}{4} & 0 & 0 & 0 & 0 \\ 0 & 0 & 0 & 0 & 0 & 0 & 0 & 0 & 0 & 0 & 0 & -\frac{14}{5} & 0 & 0 & \frac{2}{5} & -\frac{2}{5} \\ 0 & 0 & 0 & 0 & 0 & 0 & 0 & 0 & 0 & 0 & 0 & 0 & -5 & 0 & -1 & \frac{19}{5} \\ 0 & 0 & 0 & 0 & 0 & 0 & 0 & 0 & 0 & \frac{4}{5} & 0 & -\frac{4}{5} & 0 & -\frac{16}{5} & 0 & 0 \\ 0 & 0 & 0 & 0 & 0 & 0 & 0 & 0 & 0 & 0 & 0 & 0 & 0 & 0 & -\frac{7}{2} & \frac{1}{10} \\ 0 & 0 & 0 & 0 & 0 & 0 & 0 & 0 & 0 & 0 & 0 & 0 & 0 & 0 & \frac{1}{10} & -\frac{7}{2} \end{matrix} \right]$.

From Eq. (S4), we obtain sixteen distinct eigenvalues $({0.67}^{q}, 0.45, 0.37, 0.30, 0.25, 0.20, 0.17, 0.14, 0.11, 0.09, 0.07, 0.06, 0.05, 0.04, 0.033, 0.027)$ by taking $\Delta t=1$. It is obvious that ${0.67}^{q}\to1$ as $q\to0$, indicating that there is a critical value $q_{c}=0$, where the system undergoes a critical transition. We aimed to detect early warning signals that indicate the critical transition as a control parameter $q$ approaches a critical value 0.

It should be noted that the kinetic model has nothing to do with the application on real biological data. Model Eq. (S2) is only for a numerical simulation that is totally irrelevant with the real-data applications.

## Supplementary Note S3. One-sample *t*-test

The one-sample *t*-test [17] is employed to determine whether constant $z$ is statistically significantly different from the mean of an $n$-dimensional vector $\boldsymbol{Z}=(z_{1},z_{2},\cdots,z_{n})$. The one-sample *t*-test statistic is defined by the following equation:

$ST=\frac{\bar{Z}-z}{SD(\boldsymbol{Z})/\sqrt{n}}$ (S5)

where $\bar{Z}$ represents the mean of vector $\boldsymbol{Z}$ and $SD(\boldsymbol{Z})$ represents the standard deviation of vector $\boldsymbol{Z}$. The statistical index $ST$quantitatively measures the significant difference between $\bar{Z}$ and $z$. To estimate the statistical significance, the P-value $P$ (the probability associated with $ST$) can be obtained by the $t$-distribution. There is a significant difference between $\bar{Z}$and $z$ if $P<0.05$, otherwise, the difference is not significant. In this study, to accurately analyze the dynamic process of complex diseases based on the ERE index, we utilize the above strategy to confirm the appearance of the critical point. The time point $T=t$ is considered to be a critical point if there is a significant difference between the ERE index $H(t)$ and the mean value of a vector $(H\left( 1 \right),H\left( 2 \right),\ldots,H\left( t-1 \right))$ ($P<0.05$). Specially, when $t=2$, the time point $T=t$ is considered a critical point if $H\left( t \right)$ is significantly different from the mean of vector $(H(1), H(3))$.

## Supplementary Note S4. Summary for positive and negative edges of TCGA datasets

As shown in the Table S4, a number of gene pairs that are strongly linked to patient prognosis are included in the signaling gene pairs for each dataset. For a pair of genes, if patients with high-entropy gene pairs (top 5%) containing it have significantly ($P\leq0.05$) longer/shorter overall survival times compared to patients with high-entropy gene pairs where this specific gene pair isn't present, it is termed a positive/ negative edge biomarker. It is easy to note that the majority of gene-gene interactions have a negative effect on patient prognosis.

## Supplementary Note S5. Verification for the identified critical state

To verify the identified critical state, we used a procedure [18] as shown in Fig. S3. For example, if ERE determines that the critical stage is IIA, then the first survival test compares samples from the before-transition period (IA-IIA) with those from the after-transition period (IIIB-IV), the second survival test compares any two stages from the before-transition except for the critical stage, the third survival test compares any two stages from the after-transition except for the critical stage, and the fourth survival test compares the critical point (IIA) with the following stage of it (IIB). In addition, when survival test 1 and test 4 have a significant difference in survival time, it indicates that the identified critical stage is highly related to prognostic survival time. Furthermore, when test 2 and test 3 have a negligible difference in survival time, it indicates that no other stage is related to the critical transition in survival time. A diagram of the validation process is displayed in Supplementary Figure S3. The results of applying the procedure to THCA and KIRP are shown in Supplementary Figure S4.

ERE determined stage II to be the critical state for THCA. There is a significant difference between the survival time of samples from the before-transition stage (stages I–II) and those from the after-transition stage (stages III–IV) ($P<0.0001$; Fig. S4A). Specially, survival time for patients in stages following the critical shift is shorter than that for those in the stage prior to the transition. Stage I-II samples have a considerably longer life duration than stage III samples, merely considering samples near the critical stage ($P=0.0018$; Fig. S4B). Survival analysis of after-transition samples has also been done to see whether there is any other important transition that causes a difference in survival time. Specifically, survival curves from samples in stages III and IV did not differ significantly ($P=0.36$; Fig. S4C) (the after-transition stage).

For KIRP, stage III was identified as the critical stage by ERE. As shown in Fig. S4D, there is a significant difference ($P<0.0001$) between the survival curves of samples before and after stage III of KIRP. Clearly, the survival time of before-transition samples (samples from stages I-III) is significantly longer than that of after-transition samples (samples from stages IV). In addition, there is a statistically significant difference ($P=0.032$; Fig. S4E) between the survival curves at the critical point (stage III) and its subsequent stage (IV). However, the survival curves of samples from pre-transition stages (stage I and stage II) differ only marginally ($P=0.9$; Fig. S4F), confirming that there is no additional essential transition that results in survival curve differences.

In addition, we performed bootstrapping on 95% of the samples in each stage (including the reference samples) to calculate the ERE scores and repeated the procedure 500 times. The results indicate that, among these 500 experiments, the repeatedly appearing (with an occurrence rate above 95%) local maximum ERE scores corresponding to stage IIB in COAD, stage IIIB in LUAD, stage II in THCA, and stage II in KIRC, respectively, demonstrate statistical validation for the analysis results of the manuscript, as shown as in the Fig. S15 and Table S5.

## Supplementary Note S6. ERE gene pairs affect the Rap1 signaling pathway in tumor progression

The signaling genes from the critical state in KIRC and related DEGs were significantly enriched in Rap1 signaling pathway which playing a critical role in tumor cell migration and invasion [19, 20]. NMDARs are coded by *GRIN2A* and *GRIN2B* [21], which is precisely a signaling gene pairs with high ERE score, which means that the receptor receives signals from the microenvironment. Through Cam and Epac, signals further activate *Rap1*, *SRC*, *MAP2K6* which affects proliferation ability and cell adhesion in tumor cells after the tipping points. However, *Rap1* was always at low expression level, the ERE molecules and related DEGs provoke a downstream cascade reaction and play an essential role in tumor progression (Fig. S7).

## Supplementary Note S7. KEGG pathway enrichment analysis

KEGG (Kyoto Encyclopedia of Genes and Genomes) database is a database that systematically analyzes gene functions, linking genomic information and functional information, including the PATHWAY database, GENES database, GENOME database, etc. The Pathway database is the most widely used public database of metabolic pathways [22-24].

Here, the pathway enrichment indicate that the proportion of genes annotated to a certain metabolic pathway in the selected gene group is significantly larger than that of the background genes annotated to the metabolic pathway in all background genes. Therefore, pathway enrichment analysis involves foreground genes and background genes, where the foreground genes are the gene set that you focus on, and the background genes refer to all the genes of the species. The hyper-geometric test [25] is employed to test whether a pathway is significantly enriched by the signaling genes.

The enrichment significance ($P$-value) can be defined as follows:

$P=1-\sum_{i=0}^{m-1} \frac{\binom{M}{i}\binom{N-M}{n-i}}{\binom{N}{n}}$, (S6)

where $N$ is the number of genes with pathway annotation among all genes; $n$ is the number of signaling genes in $N$; $M$ is the number of genes annotated to a specific pathway among all genes; $m$ is the number of signaling genes annotated to the specific pathway.

The calculated $P$ value is further corrected by multiple testing to obtain a corrected-p value (*i.e.*, $Q$-value). Generally, we set $Q$-value$\leq0.05$ as the threshold, and the pathways meeting this condition are defined as the significantly enriched ones by signaling genes.

## Supplementary Note S8. Theoretical basis

The theoretical background is the DNB theory. Specifically, in order to theoretically and mathematically describe the dynamics of a complex disease, its evolution is usually modeled as a time-dependent nonlinear dynamical system [26], in which the sudden deterioration is regarded as a state transition at a bifurcation point [27]. Generally, the after-transition state refers to a stable stage after a drastic deterioration. Thus, the after-transition state may be characterized by different deterioration processes (as shown in Fig. S16). During the progression of cancer, for instance, it is regarded that the stage of lymphoid infiltrates as the after-transition state after a critical transition of tumor cell invasion. On the other hand, after a critical transition of metastasis, the after-transition state may be taken to represent the stage of being distant metastasis. In ideal situation with small noise, when a complex system is near the critical point, among all observed variables there exists a dominant group defined as the DNB biomolecules, which satisfy the following three conditions based on the observed data [28]:

1. The correlation (${PCC}_{\mathrm{in}}$) between any pair of members in the DNB group rapidly increases;
2. The correlation (${PCC}_{\mathrm{out}}$) between one member of the DNB group and any other non-DNB member rapidly decreases;
3. The standard deviation (${SD}_{\mathrm{in}}$) or coefficient of variation for any member in the DNB group drastically increases.

The above three properties are necessary conditions of the state transition at a codimension-one bifurcation point, and can also be approximately stated as: the occurrence of a group of biomolecules whose expressions are strongly fluctuating and highly correlated, implies an upcoming critical transition. These three properties are the theoretical basis of DNB method. From the above three properties, it is clear that the critical transition of a system is actually indicated by “the transition of distribution”, that is, for some variables (DNB members), their expression distribution would significantly change when the system approaches the critical transition point. Therefore, by exploring the differential distributions (rather than differential expressions) of some variables, it is possible to identify the upcoming qualitative state transition (Fig. S17).

The basic definition of relative entropy is a valuable tool for comparing and quantifying the differences between probability distributions, which can be applied to various biological data analysis tasks, such as gene expression analysis [29], sequence alignment [30], functional annotation [31]. However, it cannot detect dynamic changes at the individual level in gene interactions. Therefore, our method draws upon and adapts the standard representation of relative entropy, resulting in a novel metric that provides a new perspective and tool for individual-level network analysis. Specifically, KDE is advantageous for estimating unknown distributions from empirical data and providing density calculations for any shape without being affected by grid size or location effects [32]. Therefore, we evaluate a smooth empirical probability density function (pdf) based on individual locations of all sample data through KDE. However, bandwidths in KDE are calculated from the observed data reflecting the current characteristics of the system. Therefore, we calculate the “relative entropy” by only evaluating at the sampled points. Furthermore, if we evaluate $p_{r}$ and $p_{c}$ at the same value within the sum, we can only obtain a scalar representing the overall expression distribution difference, failing to capture the individual-level changes in gene interactions and cannot detect samples in a critical state. Therefore, we modified the standard representation of relative entropy by evaluating $p_{r}$ and $p_{c}$ at different points to quantify the individual-level dynamic changes. Taking into account the above considerations and experimental results, our approach shows several advantages. Compared to common node-based methods, ERE is more sensitive to early-warning signals, displaying robustness against sample number and noise. Additionally, it offers a promising way to identify critical transitions in complex diseases from a gene-pair perspective, enabling the tracking of dynamic changes in cooperative molecular associations.

## Supplementary Note S9. Performance of ERE in numerical simulation

A simulated sixteen-node model network, which represents a regulatory network with a system of stochastic differential equations (Equation S2) shown in Supplementary Information (see Fig. S9 and Supplementary Note S2 for details), was employed to validate the proposed ERE algorithm. Such a regulatory network model of the Michaelis‒Menten form is generally utilized for research about genetic regulation behavior, including transcription, translation [33-35], diffusion, and other nonlinear biological progressions [36]. Moreover, the state transition in gene regulatory networks is usually considered as a bifurcation of Michaelis-Menten form in the dynamical system [10]. On the basis of a varying parameter $q$ ranging from −0.35 to 0.3, a custom dataset was created for the numerical experiment, wherein $q = 0$ is the bifurcation point. The samples at the initial moment ($q=-0.35$) are employed as the reference samples.

As demonstrated in Fig. S14A, a significant uplift of the ERE score signals the forthcoming critical transition as the system approaches the bifurcation parameter value $q = 0$. However, when the system is at a stable before-transition or after-transition state, *i.e.*, out of the vicinity of the bifurcation point, the ERE value is at a low level. In fact, in light of the key points in DNB theory [37], when the living system is near a bifurcation point, the expression of fractional genes fluctuates wildly. Thus, the probabilities fitted based on samples derived near the bifurcation point are significantly different from those fitted from samples of a stable state (such as the before-transition or after-transition state). The details of theoretical background are described in Supplementary Information Note S8. As illustrated in the numerical experiment, the ERE method is capable of utilizing high-dimensional information and accurately signaling the tipping point wherein the system goes through a critical transition. Furthermore, to illustrate the robustness of ERE, it was performed under (1) different number of samples and (2) different strengths of white noise. The result suggests that the ERE method still provides stable and effective signals for the tipping point (Fig. S5 and Fig. S6), indicating the robustness of ERE compared to other methods.

## Supplementary Note S10. Details for the expression calculation of DEGs

The average expression of DEGs represents the aggregate of expression of DEGs across all samples within a time point $T=t$. Specifically, for the gene expression matrix of case samples at time point $T=t$, we conducted differential analysis by comparing them with the reference samples, generating a set of differentially expressed genes (DEGs) ($P<0.05$) at time point $T=t$, denoted as $D^{t}$. Subsequently, we filtered out the top 5% significantly differentially expressed genes from $D^{t}$, denoted as $D_{top}^{t}$. The size of $D_{top}^{t}$ is denoted as $\alpha^{t}$. The case sample set at time point $T=t$ is denoted as $C^{t}$ and its size is denoted as $\beta^{t}$. Next, we computed the average value of the significantly differentially expressed genes across all samples at time point $T=t$ as follows:

$v^{t}=\frac{1}{\alpha^{t}\beta^{t}}\sum_{g\in D_{top}^{t},k\in C^{t}} \rho_{gk}^{t}$, (S7)

where $\rho_{gk}^{t}$ represents the expression value of a gene $g\in D_{top}^{t}$ in a case sample $k\in C^{t}$ at time point $T=t$. Finally, we repeated the aforementioned steps across all time points to generate the average expression data lines for DEGs presented in the main text.

## Supplementary Note S11. Details for the applied issue of ERE

Our study is retrospective in nature, aiming to detect critical points in the disease progression from the acquired time-course data. It serves as an exploratory tool to uncover significant biomolecular interactions related to "critical slowing down" but is not designed as a real-time diagnostic tool. Additionally, to our knowledge, the specific time for sample collection and the acquisition of computable data falls under patient privacy and is typically challenging to obtain. Therefore, we cannot compare the precise time course of the ERE jump with the current time lag between sample collection and the obtention of computable data. To determine the optimal edges, we tested the impact of selecting high-entropy edges at different thresholds on the detection of critical points, using both a simulated dataset and a real dataset (THCA). As shown in Fig. S20, ERE successfully identified critical points under different thresholds (top 5%, 10%, 20%, and 50%) that were employed for defining high-entropy edges, demonstrating negligible impact of thresholds on the performance of ERE.

Besides, we compare the ERE approach with pure physical approaches (*i.e.*, Recurrent Neural Network (RNN) and its refinement Long Short-Term Memory (LSTM) network) [38, 39] on the above datasets. RNNs are a type of artificial neural network designed for sequential data processing. Unlike traditional feedforward neural networks, RNNs have connections that form directed cycles, allowing them to capture temporal dependencies in data sequences. LSTM is a type of recurrent neural network architecture designed to overcome the vanishing gradient problem that occurs in traditional RNNs. LSTMs are capable of learning long-term dependencies in sequential data by maintaining an internal memory cell and a set of gates that regulate the flow of information. We have devised the following strategy to adapt RNN and LSTM for critical point detection.

First, samples at time point $T=t$ are labeled as critical state, while samples from other time points are labeled as non-critical state. Then, we randomly selected 70% of all samples as the training set to train the RNN or LSTM, with the remaining samples used as the testing set. Subsequently, we employed the trained model to classify the samples on the testing set, yielding its classification accuracy. Finally, we repeated the same procedure for the samples at each of the other time points to obtain accuracy at each time point. The time point with the highest accuracy is considered as the critical point based on the specificity of samples in the critical state.

The results depicted in Fig. S21 demonstrate that ERE provides clearer and more effective early warning signals for state transitions in both simulated and real datasets. Notably, ERE does not require model training or much parameter tuning, emphasizing its simplicity and efficiency in detecting critical points in the disease progression.

Thermodynamic entropy is a measure of disorder, expressed by

$S=k_{B}\ln W$, (S8)

where $W$ is the number of microscopic states relating to a given macroscopic thermodynamic state and $k_{B}$ is the Boltzmann constant [40]. From general physical principles, life should remain in a low-entropy state [41]. Essentially, the information quantity is the projection of thermodynamic entropy in microscopic phase-space to the subspace spanned by macroscopic states [42]. Therefore, the thermodynamic entropy of a cancerous cell is different from that of a normal cell due to the more disordered structure of the cancerous cell (such as the loss of compartmentation). Entropy production due to various dissipation mechanisms based on temperature differences, chemical potential gradient, chemical affinity, viscous stress and exerted force is a promising tool for calculations relating to potential targets for tumor isolation and demarcation [40]. Furthermore, the entropy of a system (*e.g.*, a normal cell or cancerous cell) changes with time, obeying the continuity equation (entropy balance equation) [43, 44]:

$$\frac{dS}{dt}=\int\sigma_{s}d\tau+\left( net rate of entropy flow through boundary \right)$$

$=\int\sigma_{s}d\tau+\left( entropy flow rate in \right)-(entropy flow rate out)$, (S9)

where $\sigma_{s}$ is the rate of entropy production in unit volume. Following the second law of thermodynamics, the entropy production is always positive [45]. Only when the entropy production is canceled by the outward entropy flow can the system remain in an ordered low-entropy state [46, 47]. The difference between normal and cancerous cells can be studied from the aspect of entropy production [48]. We have consulted a number of literatures [40, 42, 49, 50], to our knowledge, the rate of entropy production by a cancerous cell is always higher than that of a healthy cell apart from the case of the application of external energy. To sum up, thermodynamic entropy provides a new insight into cancer therapy, with potential as an effective tool for cancer diagnosis and staging.

On the other hand, our underlying theoretical background is the dynamic network biomarker (DNB) method, which employs the fluctuation (i.e., the standard deviation) and covariance of samples to identify the tipping point of the disease process. Specifically, when a complex biological system is near the critical point, there exists a dominant group defined as the DNB biomolecules among all observed variables (genes), satisfying the following three conditions [28]:

1. The correlation (${PCC}_{\mathrm{in}}$) between any pair of members in the DNB group rapidly increases;
2. The correlation (${PCC}_{\mathrm{out}}$) between one member of the DNB group and any other non-DNB member rapidly decreases;
3. The standard deviation (${SD}_{\mathrm{in}}$) or coefficient of variation for any member in the DNB group drastically increases.

Notably, the samples in the critical state demonstrate a more disordered expression profile of DNB molecules compared to samples from the before-transition and after-transition states, indicating the increase of disorder of the system. Consequently, the differences in probability distribution among interacting DNB molecules significantly increase at this moment. Inspired by relative entropy in information theory, we integrated the computational strategy of informational entropy into the ERE method to obtain a sample-specific quantitative metric for identifying the critical state transition of the system. Integrating ERE with thermodynamic entropy provides a more robust tool for cancer treatment, offering enhanced insights into therapeutic strategies.

# Supplementary references

1. Ho YJ, Lin YM, Huang YC, Shi B, Yeh KT, Gong Z, et al. Prognostic significance of high YY1AP1 and PCNA expression in colon adenocarcinoma. Biochem Biophys Res Commun. 2017;494:173-180.

2. Zhang XZ, Chen MJ, Fan PM, Jiang W, Liang SX. BTG2 Serves as a Potential Prognostic Marker and Correlates with Immune Infiltration in Lung Adenocarcinoma. Int J Gen Med. 2022;15:2727-2745.

3. Jonklaas J, Murthy S, Liu D, Klubo-Gwiezdzinska J, Krishnan J, Burman KD, et al. Novel biomarker SYT12 may contribute to predicting papillary thyroid cancer outcomes. Future Sci OA. 2018;4:FSO249.

4. Meng X, Yuan H, Li W, Xiao W, Zhang X. Biomarker screening and prognostic significance analysis for renal cell carcinoma. Int J Gen Med. 2021;14:5255-5267.

5. Kim J, Scott CD. Robust kernel density estimation. J Mach Learn Res. 2012;13:2529-2565.

6. Węglarczyk S. Kernel density estimation and its application. ITM Web Conf. 2018;23:00037.

7. Terrell GR, Scott DW. Variable kernel density estimation. Ann Stat. 1992;20:1236-1265.

8. Silverman BW. Density estimation for statistics and data analysis. Routledge; 2018.

9. Turlach BA. Bandwidth selection in kernel density estimation: A review. CORE Inst de Stat. 1993;19:1-33.

10. Liu R, Chen P, Chen L. Single-sample landscape entropy reveals the imminent phase transition during disease progression. Bioinformatics. 2020;36:1522-1532.

11. Chen L, Wang R, Li C, Aihara K. Modeling Biomolecular Networks in Cells: Structures and Dynamics. Springer Science & Business Media; 2010.

12. Chen L, Wang RS, Zhang XS. Biomolecular networks: methods and applications in systems biology. John Wiley and Sons; 2009.

13. Becskei A, Serrano L. Engineering stability in gene networks by autoregulation. Nature. 2000;405:590-593.

14. Chen L, Aihara K. Stability of genetic regulatory networks with time delay. IEEE Trans Circ Syst. 2002;49:602-608.

15. Li C, Chen L, Aihara K. Stability of genetic networks with SUM regulatory logic: Lur'e system and LMI approach. IEEE Trans Circuits Syst Part I Regul Pap. 2006;53:2451-2458.

16. Platen E. An introduction to numerical methods for stochastic differential equations. Acta Numer. 1999;8:197-246.

17. Rochon J, Kieser M. A closer look at the effect of preliminary goodness‐of‐fit testing for normality for the one‐sample t‐test. Br J Math Stat Psychol. 2011;64:410-426.

18. Zhong J, Liu R, Chen P. Identifying critical state of complex diseases by single-sample Kullback–Leibler divergence. BMC Genom. 2020;21:1-15.

19. Bailey CL. The roles of Rap1 in cancer metastasis and pancreatic islet beta cell function. Duke Dissertation, 2009.

20. Zhang YL, Wang RC, Cheng K, Ring BZ, Su L. Roles of Rap1 signaling in tumor cell migration and invasion. Cancer Biol Med. 2017;14:90.

21. Endele S, Rosenberger G, Geider K, Popp B, Tamer C, Stefanova I, et al. Mutations in GRIN2A and GRIN2B encoding regulatory subunits of NMDA receptors cause variable neurodevelopmental phenotypes. Nat Genet. 2010;42:1021-1026.

22. Kanehisa M, Goto S. KEGG: kyoto encyclopedia of genes and genomes. Nucleic Acids Res. 2000;28:27-30.

23. Ogata H, Goto S, Fujibuchi W, Kanehisa M. Computation with the KEGG pathway database. Biosystems. 1998;47:119-128.

24. Kanehisa M, Goto S, Sato Y, Furumichi M, Tanabe M. KEGG for integration and interpretation of large-scale molecular data sets. Nucleic Acids Res. 2012;40:D109-D114.

25. Chen J, Li C, Zhu Y, Sun L, Sun H, Liu Y, et al. Integrating GO and KEGG terms to characterize and predict acute myeloid leukemia-related genes. Hematology. 2015;20:336-342.

26. Chen P, Li Y, Liu X, Liu R, Chen L. Detecting the tipping points in a three-state model of complex diseases by temporal differential networks. J Transl Med. 2017;15:217.

27. Scheffer M, Carpenter S, Foley JA, Folke C, Walker B. Catastrophic shifts in ecosystems. Nature. 2001;413:591-6.

28. Chen L, Liu R, Liu Z-P, Li M, Aihara K. Detecting early-warning signals for sudden deterioration of complex diseases by dynamical network biomarkers. Sci Rep. 2012;2:342.

29. Yan X, Deng M, Fung WK, Qian M. Detecting differentially expressed genes by relative entropy. J Theor Biol. 2005;234:395-402.

30. Li C, Wang J. Relative entropy of DNA and its application. Physica A. 2005;347:465-471.

31. Vacic V, Uversky VN, Dunker AK, Lonardi S. Composition Profiler: a tool for discovery and visualization of amino acid composition differences. BMC Bioinformatics. 2007;8:1-7.

32. Wang Z, Ginzler C, Waser LT. Assessing structural changes at the forest edge using kernel density estimation. Forest Ecol Manag. 2020;456:117639.

33. Cantone I, Marucci L, Iorio F, Ricci MA, Belcastro V, Bansal M, et al. A yeast synthetic network for in vivo assessment of reverse-engineering and modeling approaches. Cell. 2009;137:172-181.

34. Hogenesch JB, Ueda HR. Understanding systems-level properties: timely stories from the study of clocks. Nat Rev Genet. 2011;12:407-416.

35. Sherman MS, Cohen BA. Thermodynamic state ensemble models of cis-regulation. PLoS Comput. Biol. 2012;8:e1002407.

36. Alnahhas RN, Sadeghpour M, Chen Y, Frey AA, Ott W, Josić K, et al. Majority sensing in synthetic microbial consortia. Nat Commun. 2020;11:3659.

37. Chen P, Liu R, Li Y, Chen L. Detecting critical state before phase transition of complex biological systems by hidden Markov model. Bioinformatics. 2016;32:2143-2150.

38. Elman JL. Finding structure in time. Cogn Sci. 1990;14:179-211.

39. Hochreiter S, Schmidhuber J. Long short-term memory. Neural Comput. 1997;9:1735-1780.

40. Luo L, Molnar J, Ding H, Lv X, Spengler G. Physicochemical attack against solid tumors based on the reversal of direction of entropy flow: An attempt to introduce thermodynamics in anticancer therapy. Diagn Pathol. 2006;1:1-7.

41. Schrödinger E. What is life? The physical aspect of the living cell and mind. Cambridge university press Cambridge; 1944.

42. Luo L, Molnar J, Ding H, Lv X, Spengler G. Ultrasound absorption and entropy production in biological tissue: A novel approach to anticancer therapy. Diagn Pathol. 2006;1:1-6.

43. Mishin Y. Thermodynamic theory of equilibrium fluctuations. Ann Phys. 2015;363:48-97.

44. Aristov VV, Buchelnikov AS, Nechipurenko YD. The use of the statistical entropy in some new approaches for the description of biosystems. Entropy. 2022;24:172.

45. Alonso AA, Ydstie BE. Process systems, passivity and the second law of thermodynamics. Comput Chem Eng. 1996;20:S1119-S1124.

46. Aoki I. Entropy production in human life span: A thermodynamical measure for aging. Age. 1994;17:29-31.

47. Sabater B. Entropy perspectives of molecular and evolutionary biology. Int J Mol Sci. 2022;23:4098.

48. Molnar J, Thornton BS, Molnar A, Gaal D, Luo L, Bergmann-Leitner ES. Thermodynamic aspects of cancer: Possible role of negative entropy in tumor growth, its relation to kinetic and genetic resistance. Lett. in Drug Design and Rec. 2005;2:429-438.

49. Molnar J, S Thornton B, Gabor P. Thermodynamics and information physics offer new opportunities in cancer therapy. Curr Cancer Ther Rev. 2014;10:234-245.

50. Ding C, Luo L. Measurement of entropy production in living cells under an alternating electric field. Cell Biol Int. 2013;37:233-238.
